# Reorienting Mechanism of Harderoheme in Coproheme Decarboxylase—A Computational Study

**DOI:** 10.3390/ijms23052564

**Published:** 2022-02-25

**Authors:** Wei Liu, Yunjie Pang, Yutian Song, Xichen Li, Hongwei Tan, Guangju Chen

**Affiliations:** Key Laboratory of Theoretical and Computational Photochemistry, Ministry of Education, College of Chemistry, Beijing Normal University, Beijing 100875, China; 201731150027@mail.bnu.edu.cn (W.L.); pangyunjie66@hotmail.com (Y.P.); 202021150073@mail.bnu.edu.cn (Y.S.); xcli@bnu.edu.cn (X.L.)

**Keywords:** targeted molecular dynamics simulation, coproheme decarboxylase, rotation, two-step decarboxylation

## Abstract

Coproheme decarboxylase (ChdC) is an important enzyme in the coproporphyrin-dependent pathway (CPD) of Gram-positive bacteria that decarboxylates coproheme on two propionates at position 2 and position 4 sequentially to generate heme b by using H_2_O_2_ as an oxidant. This work focused on the ChdC from *Geobacillus stearothermophilus* (GsChdC) to elucidate the mechanism of its sequential two-step decarboxylation of coproheme. The models of GsChdC in a complex with substrate and reaction intermediate were built to investigate the reorienting mechanism of harderoheme. Targeted molecular dynamics simulations on these models validated that harderoheme is able to rotate in the active site of GsChdC with a 19.06-kcal·mol^−1^ energy barrier after the first step of decarboxylation to bring the propionate at position 4 in proximity of Tyr145 to continue the second decarboxylation step. The harderoheme rotation mechanism is confirmed to be much easier than the release–rebinding mechanism. In the active site of GsChdC, Trp157 and Trp198 comprise a “gate” construction to regulate the clockwise rotation of the harderoheme. Lys149 plays a critical role in the rotation mechanism, which not only keeps the Trp157–Trp198 “gate” from being closed but also guides the propionate at position 4 through the gap between Trp157 and Trp198 through a salt bridge interaction.

## 1. Introduction

As the most ubiquitous member of the metalloporphyrin family, heme b is present in various proteins such as hemoglobin, myoglobin, peroxidases, cytochrome P450, NO synthase and mitochondrial complexes II and III [1,2,3]. Heme b has versatile biological functions, including binding with dioxygen to catalyze a series of intracellular reactions in the bloodstream and becoming involved in oxygen transport, oxygen reduction and electron transfer processes [4,5,6,7]. There is more than one pathway for heme b biosynthesis, including the protoporphyrin-dependent pathway (PPD) in Gram-negative bacteria and the coproporphyrin-dependent pathway (CPD) in Gram-positive bacteria [8,9]. Both the PPD and CPD pathways share a common process in the final stage to decarboxylate coproheme into heme b by employing H_2_O_2_ as the oxidant, in which coproheme is decarboxylated on two propionates at position 2 and position 4 sequentially by coproheme decarboxylase (ChdC) [10,11,12,13,14].

**Figure 1:**
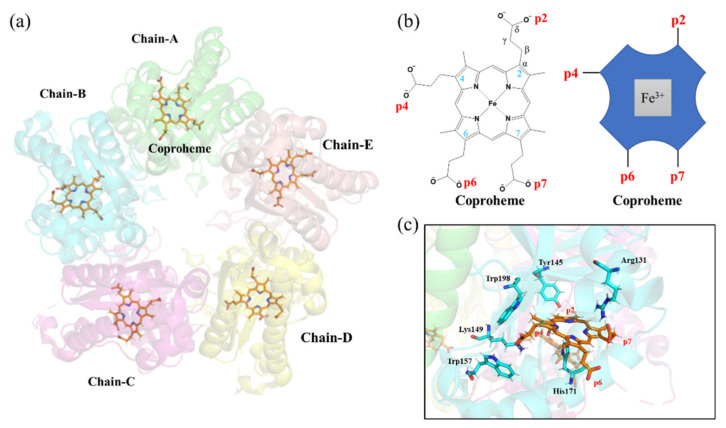
Coproheme–GsChdC complex highlighting the relevant binding orientations for the substrate. (**a**) Cartoon representation of pentamer assemblies of representative crystal structures of the coproheme–GsChdC complex: different chains of GsChdC are shown in green for chain A, cyan for chain B, magenta for chain C, yellow for chain D and wheat for chain E, respectively. The coproheme cofactor is depicted as orange sticks. (**b**) The chemical structure of coproheme (left) and its schematic representation (right). (**c**) Close-up view of the active site structures binding with a coproheme, highlighting the positions of the side chains for the neighboring Tyr145, Arg131, Lys149, Trp157, His171 and Trp198 in cyan sticks and coproheme in orange sticks.

Extensive research has been performed to investigate the biochemical process catalyzed by ChdC [15,16]. The crystal structures of ChdC from different resources have also been resolved. To date, more than 20 different available crystal structures of ChdCs from different bacteria have been deposited in the Protein Data Bank (www.pdb.org, 1 November 2021) [17]. In 2017, DuBois and coworkers solved the crystal structure of ChdC from *Geobacillus stearothermophilus* in a complex with the substrate analog Mn-coproporphyrin (GsChdC, PDB code: 5t2k), as shown in Figure 1a [15]. The tetrapyrrole of coproheme is slightly ruffled, with its four propionate side chains at position 2 (p2), position 4 (p4), position 6 (p6) and position 7 (p7) of each pyrrole ring, A–D, including the two reactive propionates at p2 and p4 and two unreactive propionates at p6 and p7, located below its approximate plane, as shown in Figure 1b. A histidine (His171) ligand in the proximal pocket binds to the Mn center of coproheme through the imidazole N_ε_ [15]. Several H-bond networks form around the propionate side chains of coproheme [18,19,20], such as the H-bond between p2 and Tyr145 [15]. The two bulky hydrophobic residues of Trp157 and Trp198 are located close to the p4 propionate [15]. The p4 and p7 propionate carboxyls of coproheme also establish salt bridge interactions with Arg131 and Lys149 [15], respectively, as shown in Figure 1c.

DuBois found that Tyr145 is essential for the decarboxylation function of GsChdC [15]. Our previous density functional theory (DFT) study revealed that, coupled with the O–O bond breaking of hydrogen peroxide, Tyr145 mediates the proton-coupled electron transfer (PCET) process to abstract a hydrogen atom from the p2 side chain with a free energy barrier of 19.16 kcal·mol^−1^ [21]. A very recent hybrid quantum mechanics/molecular mechanics (QM/MM) study also suggested that Tyr145 is directly involved in the decarboxylation of both p2 propionate and p4 propionate of coproheme [22]. In the crystal structure of GsChdC in a complex with coproheme, Tyr145 is located close to the p2 site to readily decarboxylate it. Since the experiment results indicated that Tyr145 also engages in p4 decarboxylation, it is rational to speculate that, after the decarboxylation of p2, harderoheme experiences reorientation to place p4 toward Tyr145. Such a process could be accomplished either through the intermediate harderoheme rotation along the Fe–N_ε(His171)_ axis or through a harderoheme release–rebinding mechanism [23,24,25]. In a recent study, by mutating His118, which is located between the p6 and p7 side chains, to a bulkier phenylalanine, Hofbauer found that the His118Phe mutant of CdChdC was unable to perform the second decarboxylation reaction of p4 [25]. However, the His118Ala mutation did not interrupt the production of heme b [25]. Hofbauer therefore suggested that Phe118 might inhibit the second decarboxylation step by hindering the reorientation process of the reaction intermediate harderoheme and proposed a rotation mechanism of harderoheme in the active site of CdChdC [25,26]. However, this model does not fully exclude the possibility that harderoheme accomplishes reorientation through a release–rebinding mechanism. There is another puzzle related to the reorientation process of harderoheme. Dubois found that, by mutating Lys149 to Ala, the consecutive reactions were stopped at the reaction intermediate harderoheme, within which only p2 was decarboxylated to a vinyl of GsChdC [15]. Given that Lys149 is located 11 Å away from Tyr145, the way Lys149 contributes to the decarboxylation of p4 remains unknown.

**Figure 2:**
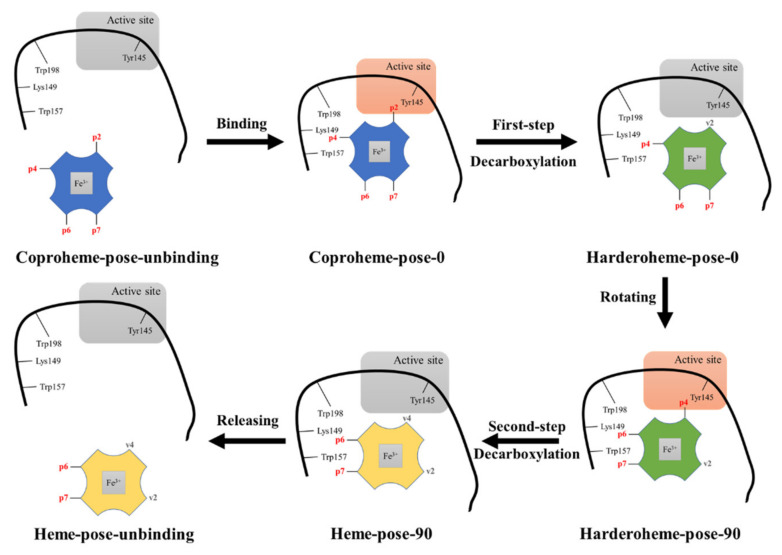
The complete catalytic process of coproheme decarboxylation of GsChdC, highlighting different relevant binding orientations of coproheme (blue), harderoheme (green) and heme b (yellow) in GsChdC with respect to the catalytic decarboxylation site Tyr145. The color of Tyr145 is switched to orange when it is ready for decarboxylation. The complete catalytic process starts with a binding process of coproheme. The propionate at position 2 (p2) is captured by Tyr145 with a hydrogen bond interaction, thereby initiating its first-step decarboxylation and the formation of vinyl (v2). The produced harderoheme rotates around the Fe–N_ε_ axis for about 90 degrees clockwise (pose 90) after losing the hydrogen bond interaction between p2 and Tyr145. The propionate at position 4 (p4) is captured by Tyr145 with a hydrogen bond interaction again, which attacks p4 for the second step decarboxylation that forms heme b. Finally, heme b is released and delivered to the cell environment.

Based on previous works, we summarize the proposed coproheme decarboxylation process catalyzed by GsChdC and display it in Figure 2. The reaction starts with the binding of coproheme in the active site of GsChdC, by which p2 propionate is captured by Tyr145 with an H-bond interaction. The homolysis of H_2_O_2_ initiates the decarboxylation reaction, which oxidates Tyr145 into a radical through a PCET process. Then, the Tyr radical abstracts a hydrogen atom from the β carbon of the propionate group and leads to its decarboxylation. The decarboxylation of p2 breaks down its H-bond interaction with Tyr145, so that the produced harderoheme is able to reorient to place its p4 propionate toward Tyr145, which initiates decarboxylation of the p4 propionate. The reaction mechanism of the first-step decarboxylation of coproheme has been extensively investigated from different perspectives thus far [13,21,22,27]. However, the reorientation mechanism of harderoheme is still unclear. There are many questions that remain elusive: (i) Does the harderoheme adopt the rotation mechanism or the release–rebinding mechanism to initiate the second-step decarboxylation on p4? (ii) How does the reaction mechanism or intermediate reorientation mechanism rationalize the role of Lys149 in the second-step decarboxylation reaction on p4? (iii) How does the enzyme only perform sequential decarboxylation on the p2 and p4 sites of coproheme but skip its p6 and p7 sites?

This study focuses on the questions listed above. To gain an in-depth understanding of the structural and dynamic basis of the rotation mechanism of the harderoheme in the active site of GsChdC for the p4 propionate decarboxylation, molecular dynamics (MD) simulations and targeted molecular dynamics (TMD) simulations with internal energy calculations were carried out to explore the energetic details of the rotation process of harderoheme in the active site. Here, we demonstrate that (1) the possible reaction mechanism provided insight by the TMD simulation of harderoheme reorientation of GsChdC, i.e., a rotation process brings p4 propionate into proximity with Tyr145 in the active site, as opposed to the release–rebinding mechanism. (2) The help of Lys149 in the rotation process of the second-step decarboxylation reaction will be clarified. This study discovers the missing piece of the puzzle of the catalytic process of ChdCs. Our results reveal both the rotation and the release–rebinding mechanisms.

## 2. Results and Discussion

### 2.1. Building and Screening the Monomer Model GsChdC–Coproheme Complex

**Figure 3:**
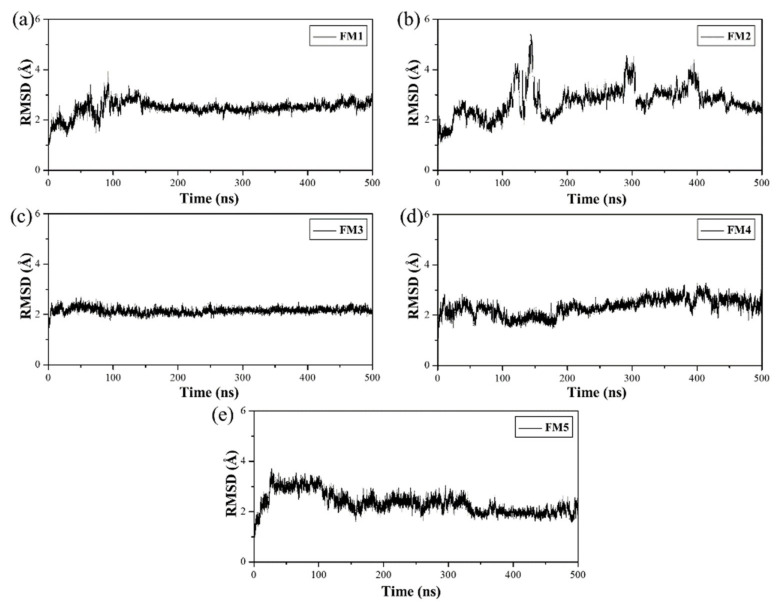
Root mean square deviation (RMSD) values of heavy atoms with respect to the initial structure of the monomer model systems, respectively: (**a**) FM1, (**b**) FM2, (**c**) FM3, (**d**) FM4 and (**e**) FM5.

**Figure 4:**
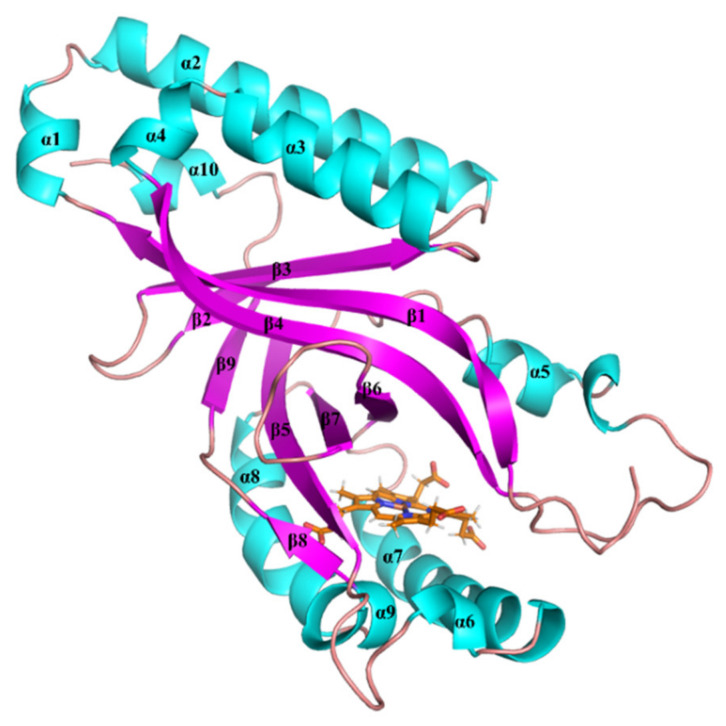
Representative structure for FM3 from the MD ensemble. The different secondary structures of the monomer model are marked as cyan for α-helix and magenta for β-sheet.

To investigate how GsChdC precisely orientates the substrate coproheme and the intermediate harderoheme to specifically decarboxylate on their p2 and p4, respectively, the five monomer models of GsChdC (from FM1 to FM5) were submitted for 500-ns MD simulation to screen an optimal model for further study. The root mean square deviation (RMSD) values of the backbone were checked to determine if the simulations achieved equilibrium. Figure 3 shows the RMSD values, which revealed that all five models attained equilibrium in 500 ns. The binding free energies, hydrogen bond and salt bridge interactions between the substrates were analyzed based on the last 100-ns MD trajectories. The detailed energy and interaction occupancy data were given in Tables S1 and S2 and Figure S2 of the Supplementary Materials. Among all five models, FM3 has the highest binding energy (−77.43 kcal·mol^−1^) between the substrate and GsChdC, with binding free energy in the five models, as shown in Table S1. DuBois suggested that residues Ser112, Tyr114, Arg131, Tyr145, Lys149, Gln185, Ser223 and Arg218 are related to the stability and reactivity of the substrate in a two-step decarboxylation reaction [15]. Table S2 shows that the total occupancies of the hydrogen bond and salt bridge interactions of these residues in FM3 are 739.72%. All these residues established hydrogen bond or salt bridge interactions with the propionate groups of the coproheme in the FM3 model. Energy decomposition shows that the energy contribution from each of these residues is almost higher than 2.00 kcal·mol^−1^. It is worth noting that, in the FM3 model, p2 propionate maintained its hydrogen bond with Tyr145 (2.1 Å) during the 500-ns simulation, which is essentially important for Tyr145 to fulfill the first decarboxylation step on p2. These results indicate that model FM3 is the most stable binding site of the substrate. Compared to the other four models, FM3 also resembles the crystal structure of GsChdC. It was thus used as the optimal model for further simulations. The representative structure of FM3 is shown in Figure 4, which is obtained from the MD simulation trajectory based on cluster analysis. The monomer model is composed of nine β-strands (β1–β9) and ten α-helices (α1–α10), as shown in Figure 4. Among them, six β-strands: β1, β4, β5, β6, β7 and β8 comprise the roof of the active site. The bottom of the active site is constructed by α7 (residues number from 162 to 179), α8 (residues number from 207 to 219) and α9 (residues number from 221 to 226). A flexible loop and the α5-helix cover the entrance of the active site. The β-strands and α-helices are linked in the order β1–α1–α2–β2–β3–α3–α4–β4–α5–β5–α6–α7–β6–β7–α8–α9–β8–β9–α10 (Figure 4).

### 2.2. Targeted Molecular Dynamics Simulation of the Reorientation Process

**Figure 5:**
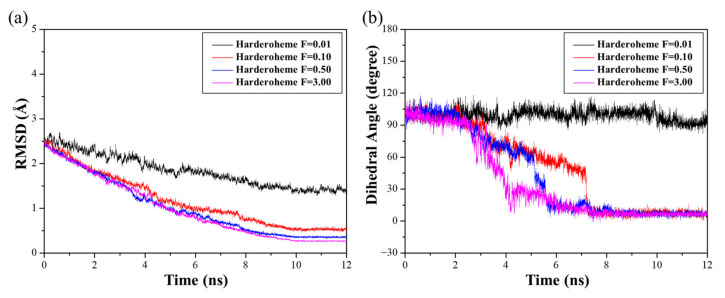
Time-dependent results from four individual targeted molecular dynamics (TMD) simulations of the harderoheme clockwise rotation process using different harmonic force constants of 0.01, 0.10, 0.50 and 3.00 kcal/(mol·Å^2^), respectively. (**a**) RMSD values of all backbone atoms. (**b**) The evolution of the dihedral angle of C_α(sub-p4)_-Fe_(sub)_-N_δ(His171)_-O_η(Tyr145)_.

As shown in Figure 2, we proposed the complete process for the two-step decarboxylation of coproheme. Although both experimental and theoretical studies confirmed that Tyr145 is responsible for decarboxylation of the p2 and p4 sites of coproheme through the PCET mechanism [21], it remains unclear how the reaction intermediate harderoheme places its p4 propionate toward Tyr145 after p2 is decarboxylated, which further raises the interesting question of how GsChdC specifically decarboxylates at the p2 and p4, but not the p6 and p7, sites of coproheme. To explore the detailed mechanism of the reorientation process of harderoheme, several models were built to investigate the possible reorientation mechanism of harderoheme in the active site of GsChdC using TMD simulations. After being decarboxylated on the p2 site, the reaction intermediate harderoheme places its p2 vinyl group adjacent to Tyr145, which we named harderoheme-pose-0. To place its p4 propionate group toward Tyr145 for the second-step decarboxylation, harderoheme needs to rotate around the Fe–N_ε(His171)_ axis approximately 90° clockwise (harderoheme-pose-90). To verify the specific decarboxylate mechanism, we examined the possibility of harderoheme rotating around the Fe–N_ε(His171)_ axis approximately 90° anticlockwise (harderoheme-pose-(−90)). For the reaction product heme b, we also examined its rotation in the active site to observe why it cannot continue to be decarboxylated on p6 sites by which heme b continues to rotate 90° clockwise (heme-pose-180). These three models (harderoheme-pose-90, harderoheme-pose-(−90) and heme-pose-180) were all submitted for 500-ns MD simulation, and the RMSD values are shown in Figure S3. The equilibrium structures were then used as the target structures for TMD simulation to simulate the rotation process of harderoheme and heme b in the active site. The TMD simulations were driven by an offset parameter based on the RMSD values. For the rotation process, the dihedral angle of C_α(sub-p4)_-Fe_(sub)_-N_δ(His171)_-O_η(Tyr145)_ was used as the rotation coordinate. The corresponding range of RMSD values related to the rotation and release–rebinding processes were calculated as below. For the rotation process, harderoheme needs to rotate clockwise approximately 90 degrees around the Fe–N_ε(His171)_ axis (from harderoheme-pose-0 to harderoheme-pose-90). To determine an appropriate harmonic force constant, for the harderoheme clockwise rotation process, 12-ns TMD simulations were performed using harmonic force constants of 0.01, 0.10, 0.50 and 3.00 kcal/(mol·Å^2^). As shown in Figure 5, the simulations with force constants of 0.01 and 0.10 kcal/(mol·Å^2^) could not reach the target. The simulations with force constants of 0.50 and 3.00 kcal/(mol·Å^2^) both reached the target with RMSD values < 0.5 Å. Meanwhile, two models were rotated around the Fe–N_ε(His171)_ axis by 92.6 and 94.7 degrees, respectively. Therefore, the TMD simulation using a force constant of 0.50 kcal/(mol·Å^2^) was used for all TMD simulations, because it is the minimum force constant required to reach the targeted structure.

#### 2.2.1. Rotation Process of Harderoheme from Pose-0 to Pose-90

We first examined the possible clockwise (from harderoheme-pose-0 to harderoheme-pose-90) and anticlockwise (harderoheme-pose-0 to harderoheme-pose-90) rotation processes of harderoheme with 12-ns TMD simulation using a full extending bias force constant of 0.5 kcal/(mol·Å^2^). The plots of the RMSD values of all the backbone atoms and variations of the rotation coordinate along the TMD simulation trajectories are shown in Figure 5 and Figure S4. To explore the energetic characteristics of the rotation process, the enthalpy was calculated by the MM-PBSA approach for the transition stage along the simulation.

**Figure 6:**
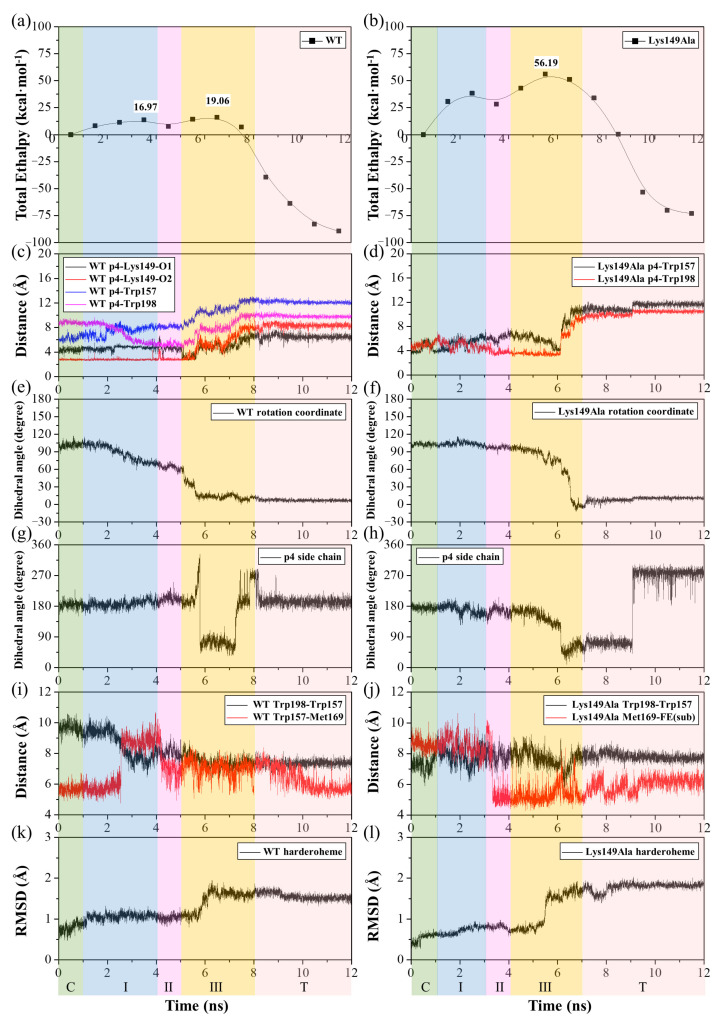
The time-dependent evolution of the harderoheme clockwise rotation of WT and the Lys149Ala mutant. (**a**,**b**) MM-PBSA enthalpy diagram of WT and Lys149Ala, respectively. (**c**) Distances between ammonium N of Lys149 and each of the two carboxylate O of p4 of WT in black and red, respectively, and distances between the C_δ_ of p4 and the mass centers of two hydrophobic residues Trp157 and Trp198 in blue and magenta, respectively. (**d**) Distances between the C_δ_ of p4 and the mass centers of two hydrophobic residues Trp157 and Trp198 in black and red, respectively. (**e**,**f**) The dihedral angle C_α(sub-p4)_-Fe_(sub)_-N_δ(His171)_-O_η(Tyr145)_ of the harderoheme-rotated angle of WT and Lys149Ala, respectively. (**g**,**h**) The dihedral angle C_α_-C_β_-C_γ_-C_δ_ of the p4 side chain of the harderoheme of WT and Lys149Ala, respectively. (**i**,**j**) Distances between the mass centers of Trp157 and Trp198 in black and between Met169 and the Fe atom in red of WT and Lys149Ala, respectively. (**k**,**l**) RMSD values of heavy atoms with respect to the initial structures of WT and Lys149Ala, respectively. The different background colors denote the different stages of the substrate rotation process: green for stage C, when the substrate is still in the initial structure stage; blue for stage I, when the substrate p4 propionate group crosses through Trp157 and Trp198 in the wild-type system but momentarily blocked in the Lys149Ala system; magenta for stage II, an intermediate stage with relatively low energy; yellow for stage III, where the salt bridges involving the p4 and p7 propionates are successively interrupted in the wild-type system, while, in Lys149Ala, the harderoheme bends under the action of Met169, and p4 manages to cross through Trp157 and Trp198 at this stage and wheat for stage T, when the substrate has evolved into the target structure.

**Figure 7:**
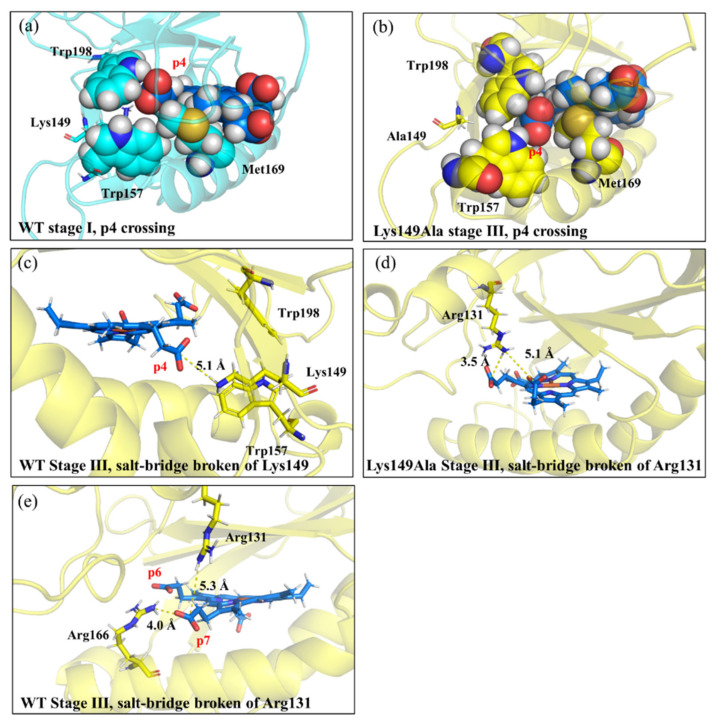
Representative active site structures at different stages of the harderoheme rotation process: (**a**) WT, stage I, p4 crossing through the Trp157 and Trp198 residues; (**b**) Lys149Ala, stage III, p4 crossing through the Trp157 and Trp198 residues; (**c**) WT, stage III, the salt bridge interruption between p4 and Lys149; (**d**) Lys149Ala, stage III, the salt bridge interruption between p4 and Arg131 and (**e**) WT, stage III, the salt bridge interruption between p7 and Arg131.

As shown in Figure 6a, for the rotation process of harderoheme from pose-0 to pose-90, the calculated energy barrier is 19.06 kcal·mol^−1^, which is a reasonable energy barrier under the experimental conditions [15]. This result agrees with the experimental result that the intermediate harderoheme can easily reorientate after the first decarboxylation step to bring its p4 propionate toward Tyr145. Based on the calculated energy curve, the rotation process of harderoheme in the active site experiences two major transition stages (stage I and stage III) and an intermediate stage (stage II) between them. Their structure snapshots, along with the initial and target structures of the TMD simulation, are shown in Figure S5A. By visually inspecting the TMD simulation trajectory, we noticed that the most important resistance of harderoheme rotation from harderoheme-pose-0 to harderoheme-pose-90 comes from the steric conflict between its p4 propionate and two bulky hydrophobic residues from Trp157 and Trp198. Moreover, the two salt bridges, the one between p4 and Lys149 and the other between p7 and Arg131, are both broken down during the harderoheme rotation process, which should also hinder the rotation. We then measured the distances between the C_δ_ of p4 and the mass center of two hydrophobic residues (Trp157 and Trp198) and the length of the salt bridge between p4 and Lys149 (measured by the N atom of Lys149 and each O atom of the p4 side chain) along the TMD trajectory and displayed them in Figure 6c. In contrast, during the whole rotation process, the distance between the p4 side chain and Trp157 keeps increasing (blue line, Figure 6c), indicating that Trp157 has no contribution to the rotation energy barrier. The steric interaction between p4 and Trp198 results in the first energy peak (stage I, 16.97 kcal·mol^−1^) in the energy curve. As shown in Figure 6c, with harderoheme beginning to rotate, the distance between p4 and Trp198 rapidly decreases (magenta line, Figure 6c) and reaches the minimum of 5.4 Å at 3.5 ns (stage I, cyan structure, Figure 7a), which corresponds to the first transition stage (stage I). Further rotation of harderoheme drives the p4 side chain to cross Trp198 at stage II (magenta structure, Figure S5), from which p4 gradually departs from Trp198 (magenta line, Figure 6c). In the meantime, the energy of the system increases again (Figure 6a). This results from breaking the salt bridge between p4 and Lys149 (black and red lines, Figure 6c). From 5 ns to 7 ns (stage III), the harderoheme rotates around the Fe–N_ε(His171)_ axis for 48 degrees. Such a motion results in an evident conformational transformation of Lys149 under the traction of the salt bridge from p4 (stage III, yellow structure, Figure 7c). As shown in Figure S6, the dihedral angle of C_γ_–C_δ_–C_ε_–C_ζ_ is used to represent the conformational change of Lys149, which converts in a range between 62 and 226 degrees from the 5-ns simulation to the 7-ns simulation. After the salt bridge is broken by crossing the 19.06-kcal·mol^−1^ energy barrier, this dihedral angle immediately turns 264 degrees (black line, Figure S6). Moreover, in the initial structure, Arg131 forms a salt bridge with p7 of harderoheme. This salt bridge is broken when the system crosses the second transition stage (stage III), by which p7 interacts with Arg166 (stage III, yellow structure, Figure 7e). From this time point, the rotation of harderoheme becomes barrierless. Such an observation clearly indicates that the p4-Lys149 salt bridge is an unfavorable factor for harderoheme rotation. However, we noticed that DuBois indicated that Lys149 is indispensable for GsChdC to decarboxylate p4 of harderoheme, which raises an interesting question of how Lys149 could facilitate the decarboxylation reaction by resisting the rotation of harderoheme.

**Figure 8:**
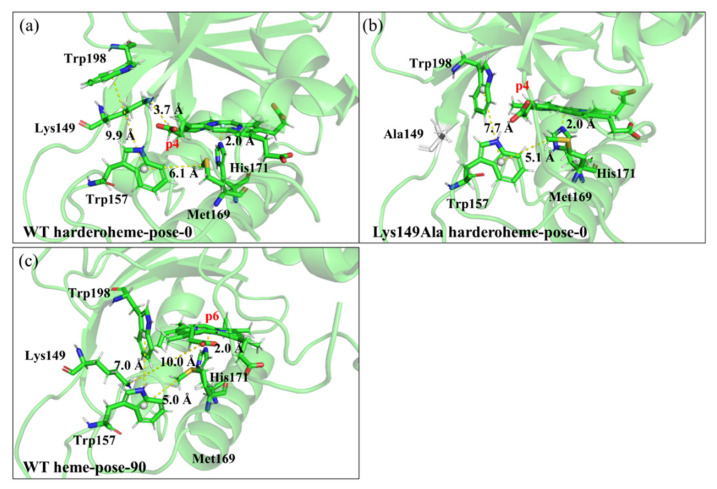
The structures after MD simulations of (**a**) wild-type GsChdC of harderoheme-pose-0, (**b**) Lys149Ala of harderoheme-pose-0 and (**c**) wild-type GsChdC of harderoheme-pose-90. Key distances among the Trp-Lys-Trp portal and the substrate are shown.

**Figure 9:**
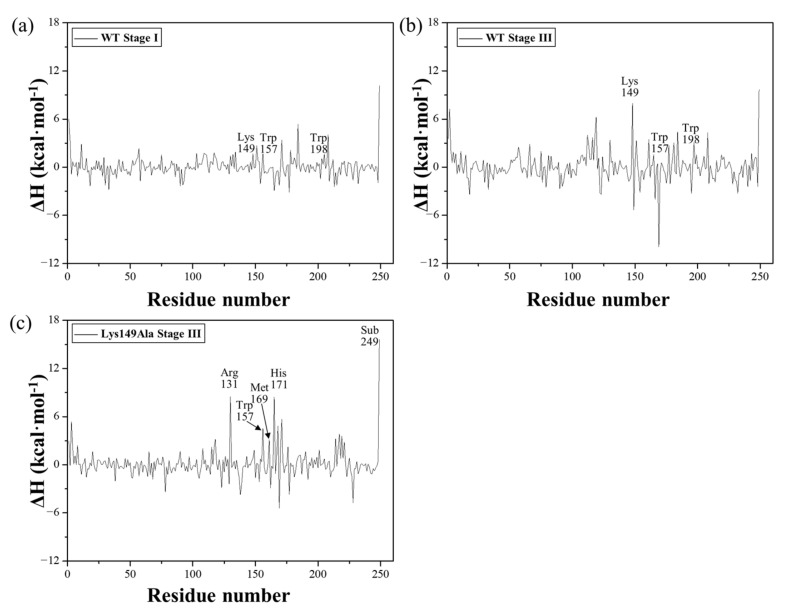
MM-PBSA energy decompositions of the barriers into residues for different stages revealed by the TMD simulations: (**a**) wild-type GsChdC, stage I; (**b**) wild-type of GsChdC, stage III and (**c**) Lys149Ala GsChdC, stage III.

To further explore whether the role of Lys149 can be rationalized by the rotation mechanism of harderoheme, we performed a TMD simulation on the rotation of harderoheme by mutating Lys149 to Ala. As shown in Figure 6b, surprisingly, the energy barrier for harderoheme rotation from pose-0 to pose-90 dramatically increases up to 56.19 kcal·mol^−1^, which means that it is practically impossible for the Lys149Ala mutant to undergo decarboxylation on p4 through a rotation mechanism. To explore the cause of such a high rotation energy barrier, we examined the equilibrium structure of the Lys149Ala mutant after the 500-ns MD simulation (shown in Figure S3C). As shown in Figure 8a,b, compared with the wild-type GsChdC, a mutation of the charged Lys149 to Ala facilitates the hydrophobic interaction between the two bulky hydrophobic residues Trp157 and Trp198, which approach each other after equilibrium MD simulation with the distance between their centers of mass shortened from 9.9 Å to 7.0 Å. Correspondingly, Trp198 moves close to the harderoheme ring and inserts between the v2 and p4 side chains, as shown in Figure 8b, which obviously blocks the pathway for p4 to rotate toward Tyr145. Their structural snapshots, along with the initial and target structures of the TMD simulation, are shown in Figure S5B. This observation was further confirmed by the TMD simulation of harderoheme rotation in the Lys149Ala mutant, which indicated that Trp157 and Trp198 are truly responsible for the very high rotation energy barrier of harderoheme in the mutant. During the first 6 ns of TMD simulation, the distance between Trp157 and Tyr198 remained less than 8 Å (black line, Figure 6d), which blocks the pathway of p4 like a closed “gate”. However, in the wild-type GsChdC, the distance between Trp157 and Tyr198 is measured as 9.9 Å in the initial structure (black line, Figure 6c). Trp157 and Tyr198 approach each other only after p4 crosses over the first peak (stage I), which allows p4 to pass through the opened “gate” between Trp157 and Trp198 with a lower energy cost. For the Lys149Ala mutant, in the initial 6 ns, the rotation of harderoheme brings its p4 toward the closed “gate” between Trp157 and Tyr198 (black and red lines, Figure 6d). The increasing steric conflict increases the energy of the system and leads to the first energy peak at 2.5 ns. The steric conflict also results in a twisted conformation of p4. The dihedral angle C_α_–C_β_–C_γ_–C_δ_ of the p4 side chain decreases from 152° to 37° (black line, Figure 6h). We noticed that, in the initial structure, Trp157 also has a hydrophobic interaction with Met169, as shown in Figure 8b. The steric conflict between p4 and Trp157 disturbs the interaction between Trp157 and Met169 and further abolishes it at 3 ns. This results in the distance between the C_ε_ of Met169 and the Fe atom of harderoheme (red line, Figure 6g). Met169 turns to interact with the harderoheme plane through a hydrophobic effect. The interaction between Met169 and harderoheme acts as an energetically favorable factor during stage II. By then, further rotation of harderoheme turns the interaction between Met169 and harderoheme into a strong steric conflict and results in a remarkable out-of-plane deformation of harderoheme (stage III, yellow structure, Figure 7b). We summarized the RMSD by harderoheme atoms in the wild-type and the Lys149Ala mutant system of the TMD simulation. In the wild-type GsChdC system, a major RMSD change is observed from the 5-ns simulation to the 7-ns simulation, by which the salt bridge breaks down so that p4 is loosened from the interaction with Lys149 and displays increased fluctuation (black line, Figure 6k). It can also be found that, during stage III of the mutant system, in which p4 passes through the Trp157–Trp198 “gate”, the RMSD value of harderoheme increases drastically due to steric conflict from Met169, which increases from 0.8 Å at 4 ns to 1.9 Å at 7 ns (black line, Figure 6l). Normal-Coordinate Structural Decomposition confirmed the saddle (B2u) deformation of harderoheme. From 4 ns to 7 ns, the estimated ∆Doop and ∆B2u values are 0.88 Å and 1.48 Å, respectively, as shown in the Table S3. In contrast, in the wild-type GsChdC, the rotation of harderoheme causes a very small structural variation (shown in Table S3). Arg131 also plays a critical role in resisting the rotation of harderoheme in the Lys149Ala mutant. In the mutant, Arg131 also undergoes a salt bridge switch from p7 to p6 during the rotation process. This switch proceeds at stage III (yellow structure, Figure 7d), which synergistically increases the second energy barrier to a very high level. We further performed energy decomposition to the energy barriers of harderoheme rotation in wild-type GsChdC and the Lys149Ala mutant, respectively. The energy decomposition results agree with our analysis of the TMD trajectory very well. As shown in Figure 9a,b, for the wild-type GsChdC, the energy decomposition indicates that Trp157 and Trp198 have very small contributions to the first energy barrier, while Lys149 plays a critical role in the second energy barrier. In the Lys149Ala mutant, as shown in Figure 9c, the four residues: Arg131, Trp157, Met169 and His171 all have major contributions to the 56.19-kcal·mol^−1^ energy barrier.

As discussed above, a steric interaction of the two bulky residues Trp157 and Trp198 is considered the major resistance against harderoheme rotation in GsChdC. The equilibrium initial structures of the Trp157Phe and Trp198Phe mutants are shown in Figure S9. Compared with the wild-type GsChdC, the tryptophan-to-phenylalanine mutation weakens the hydrophobic interaction between the two hydrophobic residues so that a gap is formed between them in both mutant models after MD simulation. In particular, in the Trp157Phe model, p4 crossed the Phe157–Trp198 gap after MD simulation. We further performed TMD calculations on the Trp157Phe and Trp198Phe mutants to examine if the rotation of harderoheme becomes easier. As shown in Figure S7B, the calculated energy barriers for harderoheme rotation from pose-0 to pose-90 in these two mutants decrease to 6.72 kcal·mol^−1^ at 3.5 ns of Trp157Phe and 16.73 kcal·mol^−1^ at 4.5 ns of Trp198Phe, which confirmed that the “gate” formed by these two tryptophan residues indeed limits the rotation movement of the substrate and the reaction intermediate. Mutating either Trp157 or Trp198 into a smaller phenylalanine results in an expansive “gate” between the two residues so that p4 propionate is able to cross through the “gate” easily.

Since GsChdC allows harderoheme to undergo clockwise rotation from pose-0 to pose-90, it is intriguing if harderoheme able to rotate anticlockwise in the active site so that p7 instead of p4 first reaches Tyr145. We therefore constructed the harderoheme-pose-(−90) model as the target structure and performed TMD simulation to obtain the trajectory from harderoheme-pose-0 to it. The anticlockwise rotation was found to be forbidden by an energy barrier as high as 92.14 kcal·mol^−1^ (8.5 ns, Figure S7A). By analyzing the TMD trajectory, we found that the anticlockwise rotation is barely blocked by the Trp157–Trp185 “gate”. However, the p7 side chain needs to pass through a rigid α-helix formed by 11 residues of α7 to approach close to Tyr145 during rotation. Furthermore, anticlockwise rotation also brings p6 close to this α-helix so that the two propionate side chains are both crowded in a narrow pocket and the interaction between two α-helices (α7 and α8) is disturbed.

#### 2.2.2. Rotation Process of Heme from Pose-90 to Pose-180

After decarboxylation of its p2 propionate, harderoheme can undergo 90° clockwise rotation to continue to decarboxylate p4 propionate. This raises another question: Why can the product heme b not keep on rotating to be further decarboxylated on its p6 propionate? We performed a TMD simulation to examine this proposed process and found that it needs to cross over a 48.57 kcal·mol^−1^ energy barrier for heme b to rotate its p6 side chain to the reaction site, which practically cannot be accomplished under physiological conditions. The initial structure of GsChdC complexed with heme b is in equilibrium by conventional MD simulation for 500 ns (shown in Figure S3), in which its p4 vinyl is located beside Tyr145 as heme-pose-180. The geometric structure of the complex shows that only Arg166 forms two bridges with the p6 and p7 side chains of heme b. Being decarboxylated on p2 and p4, heme b is unable to interact with Lys149, so it cannot stay between Trp157 and Trp198 to prevent them from closing the “gate” through hydrophobic interactions. Hence, the rotation of heme b is blocked by it, which stops p6 and p7 from passing through. The first transition stage of heme b rotation appears at 4.5 ns, when p6 is passes through the Trp157–Trp198 “gate”, as shown in Figure S10. Further rotation brings p7 to the “gate” so that the energy keeps increasing until p6 forms a salt bridge with Lys149 at 6.5 ns, which opens the “gate” so that the subsequent rotation process becomes barrierless after that. The TMD simulation confirmed that heme b is unable to rotate its p6 and p7 propionate to the active residue Tyr145 for decarboxylation.

**Figure 10:**
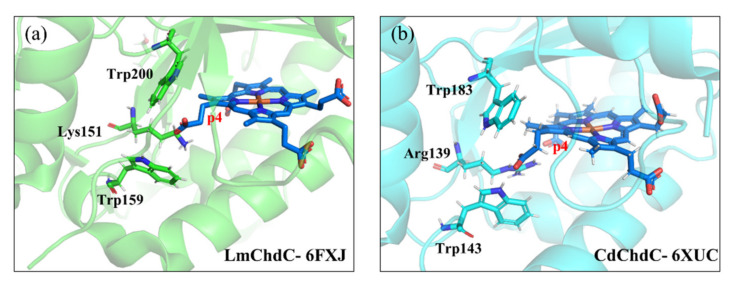
Trp–Lys–Trp portal of (**a**) LmChdC and (**b**) CdChdC.

All the TMD simulations suggest that Trp157, Trp198 and Lys149 constitute a tri-residue portal to regulate the rotation of the substrate or the intermediate in ChdC. Several recent studies resolved the structures of ChdC from different bacteria. As shown in Figure 10, LmChdC shares a similar tri-residue of the Lys151, Trp159 and Trp200 portal with GsChdC. In CdChdC, the lysine residue is replaced by an arginine, in which Arg139, Trp143 and Trp183 form the portal. These observations clearly show that the ChdC family shares a common rotation control mechanism.

#### 2.2.3. Release–Rebinding Mechanism of GsChdC

An alternative pathway for harderoheme to place its p4 propionate toward Tyr145 after the first decarboxylation reaction is the release–rebinding mechanism. TMD simulations were performed to explore whether a pathway is viable. By applying a 0.01-kcal/(mol·Å^2^) bias force constant, the release processes of harderoheme and heme b from the active site of GsChdC simulated release rebinding. As shown in Figure S7C, the energy barrier for harderoheme to be released from the active site is as high as 63.63 kcal·mol^−1^. Nevertheless, heme b only needs 20.04 kcal·mol^−1^ in energy to escape the active site. The calculation results clearly showed that the intermediate harderoheme is unable to escape from the active site so that it cannot continue to be decarboxylated on p4 through the release–rebinding mechanism. However, the final product heme b can be released from the active site easily to let the enzyme initiate a new catalytic cycle. We performed energy decomposition calculations on these two processes (shown in Figure S8). For the harderoheme-releasing process, the contribution mainly from Lys149 to the energy barrier is the most significant among all the residues due to the salt bridge formed between it and p4. The three residues Arg131, Arg151 and Gln185 also play important roles in preventing harderoheme from being released due to a salt bridge. For heme b, Lys149 cannot interact with its p4 or p2 vinyl groups, which therefore contribute little to releasing the energy barrier of heme b. Instead, the resistance for heme b release comes from the salt bridge formed by p6 and p7, which interact with Arg166 and Gln185, respectively.

## 3. Methods and Materials

The unknown structure of proteins often makes it difficult to solute intact structural information when a protein has a flexible loop structure. The coordinates of loop_112–125_ in the crystal structure of ChdC (PDB code: 5t2k) are missing. Thus, we used the MODELLER program to complete the missing loop by chain B [28,29]. The five single monomers were analyzed by MD simulation calculations. To evaluate the structural characteristics of GsChdC, the binding free energies were calculated using the MM-PBSA approach based on the corresponding MD trajectories of five single monomers. Then, the best and most stable monomer model was used to investigate the possible rotation and release–rebinding mechanisms of the substrate by TMD simulations. The initial and target structures of the TMD simulation were obtained by modifying the substrate based on that monomer model. The different substrate rotation and release–rebinding processes from the corresponding initial structure to the target structure were simulated by TMD simulation. To explore the difficulty of the different substrate reorientation process, the energy curve is indicated by the enthalpy of substrate rotation and the release–rebinding processes.

### 3.1. Model Construction for Simulations of GsChdC

The crystal structure of GsChdC in complex with Mn^II^-coproheme (PDB Code: 5t2k) was used to construct models of GsChdC. The crystal structure is in the form of homo-pentamer. In all five monomers, a flexible loop consisting of residues 112 to 125 is missing to different extents. Considering that this loop area could possibly influence substrate rotation and the release–rebinding process [15], the missing loop was built by using the MODELLER program [28,29]. In the pentamer, chain B is relatively intact, in which only residue 1 on the N-terminus and the fragment consisting of residues 114–117 were missing; it was therefore complemented using the MODELLER program. MODELLER returned five plausible completed chains, which were named FM1–FM5, as shown in Figure S1 of the Supplementary Materials. The structure coordinate as an input is an alignment of a sequence to model the missing structures with the template structures [28,29]. After modeling the missing residues, the Mn^2+^ metal center of the coproheme in each model was replaced by an Fe^3+^. For MD and TMD simulations, all model systems were explicitly solvated by using transferable intermolecular potential 3P (TIP3P) water inside a rectangular box large enough to ensure that the solvent shell was extended to 10 Å in all directions of this model [30,31]. The details of the construction processes for these models can be found in each section.

### 3.2. Molecular Dynamics Simulation

MD simulations, including minimization, heating and equilibration, were performed using the Amber20 package (version 20, URL: ambermd.org, San Francisco, CA, USA) with the classical AMBER force field ff14SB [31]. The details of the MD procedure are given in the Supplementary Materials [15,30,32,33,34,35,36,37,38,39]. The CPPTRAJ and PTRAJ utilities embedded in the Amber20 program were used to obtain the root mean square deviation (RMSD), average structures, hydrogen bond and salt bridge interactions from the trajectories [30].

### 3.3. Targeted Molecular Dynamics Simulation

Targeted molecular dynamics (TMD) was performed in the *NPT* ensemble with 1 bar pressure and 300 K temperature by the Sander module of Amber20 [40,41,42]. The following time-dependent energy function was applied in the TMD simulations:

(1) $$E_{\mathrm{TMD}}=\frac{1}{2}Nk{\left[\mathrm{RMSD}(t)-\rho \left(t\right)\right]}^{2}$$

In the equation, *N* represents the number of atoms that were used as the template. *k* is the harmonic force constant. The root mean square deviation (RMSD) of the simulated structure was used as the target index for the TMD simulation. The prescribed target RMSD value at time t is given by *ρ*(*t*), which decreases to zero linearly with time to drive the system from the initial structure to the target structure.

### 3.4. Binding Free Energy Analysis

The molecular mechanics Poisson–Boltzmann surface area (MM-PBSA) approach was used to perform the binding free energy analysis [43,44]. The binding free energy (Δ*G*_binding_) was calculated based on Equation (2):

Δ*G*_binding_ = *G*_complex_ − (*G*_ligand_ + *G*_receptor_) (2)

where *G*_complex_, *G*_ligand_ and *G*_receptor_ are the free energies of the complex, ligand and receptor calculated from the snapshots of the MD trajectories, respectively. The binding free energy was calculated as follows (2):

Δ*G*_binding_ = Δ*H*_binding_ − *T*Δ*S* (3)

Δ*G*_binding_ = ΔE_MM_ + ΔG_solv_ − TΔS (4)

The details of the MM-PBSA approach can be found in the Supplementary Materials.

### 3.5. Energetics of the Reorientation Process

The enthalpy was calculated using the MM-PBSA approach, which was used to generate the energy curve of the rotation and release–rebinding processes in this study. The MM-PBSA approach can be used to estimate the free energy of the conformational change process of proteins [45]. A previous study has shown that the MM-PBSA approach is comparable to the semiempirical quantum chemistry AM1 methods when they are employed to calculate the energy change between two intermediate conformations of 17-DMAG in a complex with human Hsp90 [46]. According to the thermodynamic expression, there is an equal relationship between the enthalpy (H) and the internal energy (U) when the pressure and volume are constant during the simulation. The enthalpy is related to the resistance of the substrate as it rotates and undergoes a release–rebinding process in the active site. Therefore, to examine the energy change during the rotation and release–rebinding processes, the total enthalpy of the protein complex during the TMD simulation was calculated using the MM-PBSA method of each conformation from 12 ns for a total of 6000 frames. The simple moving average of enthalpy was obtained by calculating the average of 500 structural enthalpies per ns. The obtained statistical average enthalpy was then plotted against its corresponding time. The entropy correction was also calculated to estimate the free energy change, as shown in Table S4.

## 4. Conclusions

In this work, we built a model of GsChdC in a complex with coproheme and investigated the reorienting mechanism of harderoheme with TMD simulations. Our calculation validated the harderoheme reorientation following a rotation mechanism with a 19.06-kcal·mol^−1^ energy barrier, which was more favorable than the release–rebinding mechanism. Our calculations revealed that Trp157 and Trp198 form a “gate” to regulate the clockwise rotation of harderoheme. Owing to the guidance from Lys149, which prevents the Trp157–Trp198 “gate” from being closed and pulls p4 propionate through the gap between Trp157 and Trp198, the clockwise rotation of harderoheme could proceed in the active site to continue the second decarboxylation reaction of p4. The role of Lys149 was further confirmed by the TMD simulation of the Lys149Ala mutant; without this critical lysine residue, Trp157 and Trp198 close the “gate” through hydrophobic interactions. Hence, p4 is blocked by steric conflict and induces Met166 to move into the proximal pocket under the harderoheme plane, which causes severe saddle deformation of the harderoheme and results in a very high energy barrier to prevent harderoheme rotation in the mutant. Such a calculation rationalizes the rotation mechanism and explains the role of Lys149. A similar Trp–Lys–Trp portal is also found in ChdC from different resources, which evidently supports the calculation results from this work. Anticlockwise rotation of harderoheme was also examined by TMD calculations and was confirmed to be forbidden, which suggested that the rotation of the substrate and the reaction intermediate in the active site of GsChdC is a highly controlled process.

## Data Availability

Not applicable.

## Supplementary Materials

The following supporting information can be downloaded at https://www.mdpi.com/article/10.3390/ijms23052564/s1.
