# Supplementary material for: Reorienting Mechanism of Harderoheme in Coproheme Decarboxylase—A Computational Study
Source: Int J Mol Sci. 2022 Feb 25;23(5):2564. doi: 10.3390/ijms23052564 (PMC8910490; doi:10.3390/ijms23052564)
Supplement: Supplementary file 1 [file ijms-23-02564-s001.zip › Supplementary Information-revision-2st.pdf]

Reorienting mechanism of harderoheme in coproheme decarboxylase - a computational study

Wei Liu[1], Yunjie Pang[1], Yutian Song[1], Hongwei Tan[1,*], Xichen Li[1,*], Guangju Chen[1,*]

[1]Key Laboratory of Theoretical and Computational Photochemistry, Ministry of Education, College of Chemistry, Beijing Normal University, Beijing 100875, China; 201731150027@mail.bnu.edu.cn (W.L.); pangyunjie66@hotmail.com (Y.P.); 202021150073@mail.bnu.edu.cn (Y.S.); xcli@bnu.edu.cn (X.L.)

* Correspondence: hongwei.tan@bnu.edu.cn (H.T.); gjchen@bnu.edu.cn (G.C.)

Section S1. Molecular Dynamics Simulation Protocols

The systems were minimized for six rounds with the positional restraints on the protein backbone gradually decreased as 100, 75, 50, 25, 10 and 0 kcal/(mol·$Å^2$). 1200 steepest descent steps followed by 1800 conjugate gradient steps were performed for each of the minimization round [1]. Then, the system was gradually heated from 0 to 300 K under the NVT ensemble, for 120 ps with a 2 fs time step. Thirdly, the temperature of 300 K (NPT) was kept and an equilibration for 200 ps running time was carried out at a pressure of 1 bar of each model. During this procedure, Langevin thermostat [2] with collision frequency of 2 ps^{-1} and Berendsen barostat [3] with pressure relaxation time of 1 ps were used to maintain the temperature and density of the system. During all the MD and TMD simulations, Particle Mesh Ewald (PME) [4] method with a cutoff distance of 10 Å for nonbonded interactions was used. The SHAKE [5] method was utilized to constrain the covalent bonds associated with the hydrogen atoms within the system. 24 Na^+ and 11 Cl^- counterions were added to achieve electroneutrality and to satisfy the experimental ionic strength of 50 mM for all models [6].

Section S2. MM-PBSA Calculations of Binding Free Energy

Using the MM-PBSA method in single-trajectory by Amber20, the last 100 ns trajectory of each monomer models were used to calculate the binding free energy calculations between enzyme and coproheme, namely, the 500 snapshots of each model at a 10-ps interval for computation. The energy decompositions of each residue in our models were performed on the molecular mechanics [7], solvation free energies without consideration of entropy calculations.

Section S3. Entropy Correction Calculation of Reorientation Process

Based on the energy curve calculated for the harderoheme reorientation process (the detail for energy curve calculation is provided in the main text, section 3.5), we identified the stationary points including the highest peak of each transition stages and the minimal point of each intermediate stages. Since each of these stationary points on the energy curve is averaged from a 1 ns trajectory, 20 frames were therefore extracted from the corresponding 1ns trajectory for entropy calculation by using MM-PBSA method. The obtained statistical average entropy was used to obtain the corresponding free energy for each of the stationary points as listed in Table S4**.**

Section S4. Parameters of Coproheme and Harderoheme

The force field parameters of heme (all-atom, include Fe^{3+} ion) were obtained from AMBER parameter database (http://amber.manchester.ac.uk/), which were developed by Giammona and modified for all-atom heme. Based on the parameters of propionate p6 and p7 in heme, we fitted the force field parameters for p2 and p4. The atomic charges of coproheme and harderoheme were assigned by using AM1-BCC method embedded in Antechamber module of Ambertools package [8-10]. The parameters files are given as follow:

The parameters of coproheme and harderoheme:

***.frcomd**

MASS

NP 14.01
NO 14.01
CY 12.0
CX 12.0
CD 12.0
FE 55.85
LC 12.01
LO 16.00

BOND

FE-NP	50.000	2.01000	
FE-LO	200.000	1.78	
FE-LC	200.000	1.75000	
LC-LO	1000.000	1.12000	
FE-NB	60.000	2.01000	
FE-NO	50.000	2.01000	
NP-CC	316.000	1.38400	
NO-CC	316.000	1.38400	
CC-CB	273.000	1.44400	
CC-CD	391.000	1.39100	
CB-CB	418.000	1.35700	
CB-CT	297.000	1.50100	
CB-CY	297.000	1.50100	
CD-HC	338.000	1.09000	
CY-HC	340.000	1.08000	
CX-HC	340.000	1.08000	
CY-CX	570.000	1.34000	
LO-LO	848.000	1.25000	unbound O2 ligand

ANGLE

NB-FE-NP	50.000	90.000
NB-FE-NO	50.000	90.000
NB-FE-LC	0.000	90.000
NB-FE-LO	0.000	90.000
NP-FE-NP	0.000	90.000
NO-FE-NO	0.000	90.000
NO-FE-NP	50.000	90.000
NO-FE-LC	45.000	90.000
NO-FE-LO	45.000	90.000
NP-FE-LC	45.000	90.000
NP-FE-LO	45.000	90.000

FE-NP-CC	30.000	127.400
FE-NO-CC	30.000	127.400
NO-CC-CB	70.000	110.300
NP-CC-CB	70.000	110.300
NO-CC-CD	70.000	125.500
NP-CC-CD	70.000	125.500
CC-NP-CC	70.000	105.400
CC-NO-CC	70.000	105.400
CC-CB-CB	70.000	107.000
CC-CD-CC	70.000	124.100
CB-CC-CD	70.000	125.400
CC-CB-CT	70.000	124.900
CB-CB-CT	70.000	128.200
CC-CD-HC	30.000	118.000
CR-NB-FE	0.000	110.000
CV-NB-FE	0.000	110.000
CB-CT-HC	35.000	109.500
CB-CB-CY	70.000	128.200
CB-CY-HC	35.000	120.000
CB-CY-CX	70.000	120.000
CY-CX-HC	35.000	120.000
CC-CB-CY	70.000	124.900
CX-CY-HC	35.000	120.000
CB-CT-CT	63.000	114.000
HC-CX-HC	35.000	120.000
FE-LC-LO	35.000	180.000
FE-LO-LO	35.000	135.000
LP-SH-LP	600.000	160.000

DIHEDRAL

X -NB-FE-X	1	0.000	0.000	2.000
X -NO-FE-X	1	0.000	180.000	2.000
X -NP-FE-X	1	0.000	180.000	2.000
X -NO-CC-X	4	5.700	180.000	2.000
X -NP-CC-X	4	5.700	180.000	2.000
X -CC-CB-X	4	3.150	180.000	2.000
X -CB-CB-X	4	21.500	180.000	2.000
X -CD-CC-X	4	7.900	180.000	2.000
X -CB-CT-X	1	0.000	180.000	2.000
X -CB-CY-X	4	0.000	180.000	2.000
X -CY-CX-X	4	30.000	180.000	2.000

X -FE-LO-X 1 0.0 180.0 2.0

IMPROPER

X -X -CC-X	0	1.000	180.000	2.000
X -X -CB-X	0	1.000	180.000	2.000
X -X -NP-X	0	1.000	180.000	2.000
X -X -NO-X	0	1.000	180.000	2.000

NONBON

FE	1.20000	0.05000	0.00000	
LO	1.60000	0.20000	0.00000	
LC	1.85	0.12	0.0	
NP	1.8240	0.1700		OPLS
NO	1.8240	0.1700		OPLS

Coproheme.prepin

0 0 2

This is a remark line

CEM.res

CEM INT 0

CORRECT OMIT DU BEG

0.0000

1	DUMM	DU	M	0	0	0	0.0000	0.0000	0.0000	.00000
2	DUMM	DU	M	1	0	0	1.4490	0.0000	0.0000	.00000
3	DUMM	DU	M	2	1	0	1.5220	111.1000	0.0000	.00000
19	FE	FE	M	16	14	12	2.1000	124.0000	180.0000	0.070000
20	NA	NP	S	19	16	14	2.0800	98.0000	90.0000	-0.585300
21	C1A	CC	S	20	19	16	1.3800	125.4000	90.0000	0.220600
22	C2A	CB	B	21	20	19	1.4100	109.0000	180.0000	-0.090200
23	CAA	CT	3	22	21	20	1.5100	124.0000	180.0000	-0.030200
24	HP71	HC	E	23	22	21	1.0900	109.5000	60.0000	0.063700
25	HP72	HC	E	23	22	21	1.0900	109.5000	300.0000	0.063700
26	CBA	CT	3	23	22	21	1.5400	111.0000	180.0000	-0.195400
27	HP73	HC	E	26	23	22	1.0900	109.5000	60.0000	0.032700
28	HP74	HC	E	26	23	22	1.0900	109.5000	300.0000	0.032700
29	CGA	C	B	26	23	22	1.5270	109.4000	180.0000	0.909600
30	O1A	O2	E	29	26	23	1.2600	117.2000	90.0000	-0.848800
31	O2A	O2	E	29	26	23	1.2600	117.2000	270.0000	-0.848800

32	C3A	CB	B	22	21	20	1.4100	107.0000	0.0000	-0.188800
33	CMA	CT	3	32	22	21	1.5100	125.0000	180.0000	-0.081900
34	HM81	HC	E	33	32	22	1.0900	109.5000	60.0000	0.074367
35	HM82	HC	E	33	32	22	1.0900	109.5000	180.0000	0.074367
36	HM83	HC	E	33	32	22	1.0900	109.5000	300.0000	0.074367
37	C4A	CC	S	32	22	21	1.4100	107.0000	0.0000	0.535900
38	CHB	CD	B	37	32	22	1.3700	127.0000	180.0000	-0.151600
39	HDM	HC	E	38	37	32	1.0800	120.0000	0.0000	0.159000
40	C1B	CC	B	38	37	32	1.3700	127.0000	180.0000	0.132600
41	NB	NO	E	40	38	37	1.3800	124.0000	0.0000	-0.465300
42	C3B	CB	B	40	38	37	1.4100	127.0000	180.0000	-0.075200
43	CAB	CT	3	42	40	38	1.5100	124.0000	180.0000	-0.024200
44	HP51	HC	E	43	42	40	1.0900	109.5000	60.0000	0.056200
45	HP52	HC	E	43	42	40	1.0900	109.5000	300.0000	0.056200
46	CBB	CT	3	43	42	40	1.5400	111.0000	180.0000	-0.193400
47	HP53	HC	E	46	43	42	1.0900	109.5000	60.0000	0.033200
48	HP54	HC	E	46	43	42	1.0900	109.5000	300.0000	0.033200
49	CGB	C	B	46	43	42	1.5270	109.4000	180.0000	0.906600
50	O1B	O2	E	49	46	43	1.2600	117.2000	90.0000	-0.852300
51	O2B	O2	E	49	46	43	1.2600	117.2000	270.0000	-0.852300
52	C2B	CB	B	42	40	38	1.4100	107.0000	180.0000	-0.213800
53	CMB	CT	3	52	42	40	1.5100	125.0000	0.0000	-0.071900
54	HM11	HC	E	53	43	42	1.0900	109.5000	60.0000	0.065367
55	HM12	HC	E	53	52	42	1.0900	109.5000	180.0000	0.065367
56	HM13	HC	E	53	52	42	1.0900	109.5000	300.0000	0.065367
57	C4B	CC	S	52	42	40	1.4100	107.0000	0.0000	0.445900
58	CHC	CD	B	53	52	42	1.3700	127.0000	180.0000	-0.049600
59	HAM	HC	E	58	57	52	1.0800	120.0000	0.0000	0.157000
60	C1C	CC	B	58	57	52	1.3700	130.0000	180.0000	0.060600
61	NC	NP	E	60	58	57	1.3800	124.0000	0.0000	-0.478300
62	C3C	CB	B	60	58	57	1.4100	127.0000	180.0000	-0.044200
63	CAC	CT	3	62	60	58	1.5100	124.0000	180.0000	-0.028200
64	HP81	HC	E	63	62	60	1.0900	109.5000	60.0000	0.059200
65	HP82	HC	E	63	62	60	1.0900	109.5000	300.0000	0.059200
66	CBC	CT	3	63	62	60	1.5400	111.0000	180.0000	-0.194400
67	HP83	HC	E	66	63	62	1.0900	109.5000	60.0000	0.033700
68	HP84	HC	E	66	63	62	1.0900	109.5000	300.0000	0.033700
69	CGC	C	B	66	63	62	1.5270	109.4000	180.0000	0.906600
70	O1C	O2	E	69	66	63	1.2600	117.2000	90.0000	-0.851300
71	O2C	O2	E	69	66	63	1.2600	117.2000	270.0000	-0.851300
72	C2C	CB	B	62	60	58	1.4100	107.0000	180.0000	-0.225800

73	CMC	CT	3	72	62	60	1.5100	125.0000	0.0000	-0.068900
74	HM31	HC	E	73	63	62	1.0900	109.5000	60.0000	0.061367
75	HM32	HC	E	73	72	62	1.0900	109.5000	180.0000	0.061367
76	HM33	HC	E	73	72	62	1.0900	109.5000	300.0000	0.061367
77	C4C	CC	S	72	62	60	1.4100	107.0000	0.0000	0.381900
78	CHD	CD	B	73	72	62	1.3700	127.0000	180.0000	-0.018600
79	HBM	HC	E	78	77	72	1.0800	120.0000	0.0000	0.166000
80	C1D	CC	B	78	77	72	1.3700	130.0000	180.0000	0.079600
81	ND	NO	E	80	78	77	1.3800	124.0000	0.0000	-0.465300
82	C2D	CB	B	80	78	77	1.4100	127.0000	180.0000	-0.014200
83	CMD	CT	3	82	80	78	1.5100	125.0000	0.0000	-0.061900
84	HM51	HC	E	83	82	80	1.0900	109.5000	60.0000	0.067367
85	HM52	HC	E	83	82	80	1.0900	109.5000	180.0000	0.067367
86	HM53	HC	E	83	82	80	1.0900	109.5000	300.0000	0.067367
87	C3D	CB	B	82	80	78	1.4100	107.0000	180.0000	-0.186800
88	C4D	CC	S	87	82	80	1.4100	107.0000	0.0000	0.677900
89	CHA	CD	S	88	87	82	1.3700	127.0000	180.0000	-0.268600
90	HGM	HC	E	89	88	87	1.0800	120.0000	0.0000	0.193000
91	CAD	CT	3	87	82	80	1.5100	124.0000	180.0000	-0.029200
92	HP61	HC	E	91	87	82	1.0900	109.5000	60.0000	0.082200
93	HP62	HC	E	91	87	82	1.0900	109.5000	300.0000	0.082200
94	CBD	CT	3	91	87	82	1.5400	111.0000	180.0000	-0.206400
95	HP63	HC	E	94	91	87	1.0900	109.5000	60.0000	0.025200
96	HP64	HC	E	94	91	87	1.0900	109.5000	300.0000	0.025200
97	CGD	C	B	94	91	87	1.5300	109.4000	180.0000	0.913600
98	O1D	O2	E	97	94	91	1.2600	117.2000	90.0000	-0.839800
99	O2D	O2	E	97	94	91	1.2600	117.2000	270.0000	-0.839800

```
LOOP   EXPLICIT
 NA    C4A
 FE    NB
 FE    NC
 FE    ND
 NB    C4B
 NC    C4C
 ND    C4D
 C1A   CHA
```

```
IMPROPER
 NA    C1A   C4A   FE
 NB    C1B   C4B   FE
```

```
 NC    C1C   C4C   FE
 ND    C1D   C4D   FE
 C1A   C2A   NA    CHA
 C1B   C2B   NB    CHB
 C1C   C2C   NC    CHC
 C1D   C2D   ND    CHD
 C2A   C3A   C1A   CAA
 C2B   C3B   C1B   CMB
 C2C   C3C   C1C   CMC
 C2D   C3D   C1D   CMD
 C3A   C4A   C2A   CMA
 C3B   C4B   C2B   CAB
 C3C   C4C   C2C   CAC
 C3D   C4D   C2D   CAD
 C4A   NA    C3A   CHB
 C4B   NB    C3B   CHC
 C4C   NC    C3C   CHD
 C4D   ND    C3D   CHA

DONE

STOP
```

Harderoheme.prepin

```
      0      0      2

This is a remark line
HEM.res
HEM     INT   0
CORRECT        OMIT DU     BEG
   0.0000
   1   DUMM   DU    M      0   0   0      0.0000     0.0000     0.0000    .00000
   2   DUMM   DU    M      1   0   0      1.4490     0.0000     0.0000    .00000
   3   DUMM   DU    M      2   1   0      1.5220   111.1000     0.0000    .00000
  19   FE     FE    M     16  14  12      2.1000   124.0000   180.0000  0.046000
  20   NA     NP    S     19  16  14      2.0800    98.0000    90.0000 -0.552300
  21   C1A    CC    S     20  19  16      1.3800   125.4000    90.0000  0.155600
  22   C2A    CB    B     21  20  19      1.4100   109.0000   180.0000 -0.053200
  23   CAA    CT    3     22  21  20      1.5100   124.0000   180.0000 -0.030200
  24   HP71   HC    E     23  22  21      1.0900   109.5000    60.0000  0.061200
  25   HP72   HC    E     23  22  21      1.0900   109.5000   300.0000  0.061200
  26   CBA    CT    3     23  22  21      1.5400   111.0000   180.0000 -0.199400
```

27	HP73	HC	E	26	23	22	1.0900	109.5000	60.0000	0.035200
28	HP74	HC	E	26	23	22	1.0900	109.5000	300.0000	0.035200
29	CGA	C	B	26	23	22	1.5270	109.4000	180.0000	0.909600
30	O1A	O2	E	29	26	23	1.2600	117.2000	90.0000	-0.838300
31	O2A	O2	E	29	26	23	1.2600	117.2000	270.0000	-0.838300
32	C3A	CB	B	22	21	20	1.4100	107.0000	0.0000	-0.239800
33	CMA	CT	3	32	22	21	1.5100	125.0000	180.0000	-0.039900
34	HM81	HC	E	33	32	22	1.0900	109.5000	60.0000	0.050700
35	HM82	HC	E	33	32	22	1.0900	109.5000	180.0000	0.050700
36	HM83	HC	E	33	32	22	1.0900	109.5000	300.0000	0.050700
37	C4A	CC	S	32	22	21	1.4100	107.0000	0.0000	0.495900
38	CHB	CD	B	37	32	22	1.3700	127.0000	180.0000	-0.094600
39	HDM	HC	E	38	37	32	1.0800	120.0000	0.0000	0.169000
40	C1B	CC	B	38	37	32	1.3700	127.0000	180.0000	0.087600
41	NB	NO	E	40	38	37	1.3800	124.0000	0.0000	-0.475300
42	C3B	CB	B	40	38	37	1.4100	127.0000	180.0000	-0.037200
43	CAB	CT	3	42	40	38	1.5100	124.0000	180.0000	-0.031200
44	HP51	HC	E	43	42	40	1.0900	109.5000	60.0000	0.063200
45	HP52	HC	E	43	42	40	1.0900	109.5000	300.0000	0.063200
46	CBB	CT	3	43	42	40	1.5400	111.0000	180.0000	-0.192400
47	HP53	HC	E	46	43	42	1.0900	109.5000	60.0000	0.027200
48	HP54	HC	E	46	43	42	1.0900	109.5000	300.0000	0.027200
49	CGB	C	B	46	43	42	1.5270	109.4000	180.0000	0.908600
50	O1B	O2	E	49	46	43	1.2600	117.2000	90.0000	-0.846300
51	O2B	O2	E	49	46	43	1.2600	117.2000	270.0000	-0.846300
52	C2B	CB	B	42	40	38	1.4100	107.0000	180.0000	-0.259800
53	CMB	CT	3	52	42	40	1.5100	125.0000	0.0000	-0.032900
54	HM11	HC	E	53	43	42	1.0900	109.5000	60.0000	0.042700
55	HM12	HC	E	53	52	42	1.0900	109.5000	180.0000	0.042700
56	HM13	HC	E	53	52	42	1.0900	109.5000	300.0000	0.042700
57	C4B	CC	S	52	42	40	1.4100	107.0000	0.0000	0.421900
58	CHC	CD	B	53	52	42	1.3700	127.0000	180.0000	-0.029600
59	HAM	HC	E	58	57	52	1.0800	120.0000	0.0000	0.145000
60	C1C	CC	B	58	57	52	1.3700	130.0000	180.0000	0.051600
61	NC	NP	E	60	58	57	1.3800	124.0000	0.0000	-0.449300
62	C3C	CB	B	60	58	57	1.4100	127.0000	180.0000	-0.067000
63	CAC	CY	B	62	60	58	1.5100	124.0000	180.0000	-0.108000
64	HV4	HC	E	63	62	60	1.0800	120.0000	0.0000	0.122000
66	CBC	CX	B	63	62	60	1.5400	111.0000	180.0000	-0.211000
67	HVC4	HC	E	66	63	62	1.0800	120.0000	0.0000	0.116500
68	HVT4	HC	E	66	63	62	1.0800	120.0000	180.0000	0.116500

72	C2C	CB	B	62	60	58	1.4100	107.0000	180.0000	-0.202800
73	CMC	CT	3	72	62	60	1.5100	125.0000	0.0000	-0.039900
74	HM31	HC	E	73	63	62	1.0900	109.5000	60.0000	0.045700
75	HM32	HC	E	73	72	62	1.0900	109.5000	180.0000	0.045700
76	HM33	HC	E	73	72	62	1.0900	109.5000	300.0000	0.045700
77	C4C	CC	S	72	62	60	1.4100	107.0000	0.0000	0.394900
78	CHD	CD	B	73	72	62	1.3700	127.0000	180.0000	-0.070600
79	HBM	HC	E	78	77	72	1.0800	120.0000	0.0000	0.166000
80	C1D	CC	B	78	77	72	1.3700	130.0000	180.0000	0.110600
81	ND	NO	E	80	78	77	1.3800	124.0000	0.0000	-0.459300
82	C2D	CB	B	80	78	77	1.4100	127.0000	180.0000	0.052800
83	CMD	CT	3	82	80	78	1.5100	125.0000	0.0000	-0.122900
84	HM51	HC	E	83	82	80	1.0900	109.5000	60.0000	0.106367
85	HM52	HC	E	83	82	80	1.0900	109.5000	180.0000	0.106367
86	HM53	HC	E	83	82	80	1.0900	109.5000	300.0000	0.106367
87	C3D	CB	B	82	80	78	1.4100	107.0000	180.0000	-0.196800
88	C4D	CC	S	87	82	80	1.4100	107.0000	0.0000	0.699900
89	CHA	CD	S	88	87	82	1.3700	127.0000	180.0000	-0.227600
90	HGM	HC	E	89	88	87	1.0800	120.0000	0.0000	0.286000
91	CAD	CT	3	87	82	80	1.5100	124.0000	180.0000	-0.033200
92	HP61	HC	E	91	87	82	1.0900	109.5000	60.0000	0.081200
93	HP62	HC	E	91	87	82	1.0900	109.5000	300.0000	0.081200
94	CBD	CT	3	91	87	82	1.5400	111.0000	180.0000	-0.191400
95	HP63	HC	E	94	91	87	1.0900	109.5000	60.0000	0.048700
96	HP64	HC	E	94	91	87	1.0900	109.5000	300.0000	0.048700
97	CGD	C	B	94	91	87	1.5300	109.4000	180.0000	0.893600
98	O1D	O2	E	97	94	91	1.2600	117.2000	90.0000	-0.852800
99	O2D	O2	E	97	94	91	1.2600	117.2000	270.0000	-0.852800

LOOP EXPLICIT

NA C4A
FE NB
FE NC
FE ND
NB C4B
NC C4C
ND C4D
C1A CHA

IMPROPER

NA C1A C4A FE

NB	C1B	C4B	FE
NC	C1C	C4C	FE
ND	C1D	C4D	FE
C1A	C2A	NA	CHA
C1B	C2B	NB	CHB
C1C	C2C	NC	CHC
C1D	C2D	ND	CHD
C2A	C3A	C1A	CAA
C2B	C3B	C1B	CMB
C2C	C3C	C1C	CMC
C2D	C3D	C1D	CMD
C3A	C4A	C2A	CMA
C3B	C4B	C2B	CAB
C3C	C4C	C2C	CAC
C3D	C4D	C2D	CAD
C4A	NA	C3A	CHB
C4B	NB	C3B	CHC
C4C	NC	C3C	CHD
C4D	ND	C3D	CHA

DONE
STOP

Table S1. Components of the MM-PBSA free energies (kcal·mol^{-1}) for the FM1, FM2, FM3, FM4 and FM5 models.

Model	FM1	FM2	FM3	FM4	FM5
ΔE_{ele}	1.11	-116.94	-114.32	-94.72	-131.38
ΔE_{vdw}	-55.02	-55.93	-54.19	-51.24	-57.79
ΔE_{int}	1.94	1.71	1.99	1.85	1.73
$\Delta G_{np/solv}$	-7.87	-8.14	-8.72	-8.24	-8.72
$\Delta G_{pb/solv}$	-32.78	55.91	47.62	30.51	69.25
ΔG_{solv}	-40.65	47.77	38.90	22.27	60.54
ΔG_{np}	-62.89	-64.07	-62.91	-59.48	-66.51
ΔG_{pb}	-31.67	-61.03	-66.70	-64.21	-62.12
$\Delta H_{binding}$	-92.61	-123.39	-127.62	-121.83	-126.90
$T\Delta S$	-47.42	-53.78	-50.19	-49.07	-52.05
$\Delta G_{binding}$	-45.19	-69.61	-77.43	-72.76	-74.85

$\Delta G_{np} = \Delta E_{vdw} + \Delta G_{np/solv}$, $\Delta G_{pb} = \Delta E_{ele} + \Delta G_{pb/solv}$

$\Delta H_{binding} = \Delta G_{np} + \Delta G_{pb} + \Delta E_{int}$

$\Delta G_{binding} = \Delta H - T\Delta S$

Table S2. Occupancies (%) of hydrogen bonds and salt bridges between coproheme and active center for FM1, FM2, FM3, FM4 and FM5 models.

Active Site	Residue	FM1	FM2	FM3	FM4	FM5
P2	Tyr145	87.58	97.58	97.94	/	97.98
	Arg218 (Salt)	/	100.0	99.60	/	100.0
	Ser223	72.94	/	94.24	29.26	/
P4	Lys149 (Salt)	/	36.62	95.24	/	63.48
	Ser112	83.36	95.54	99.00	/	89.80
P6	Tyr114	/	/	90.16	98.64	/
P7	Arg131 (Salt)	/	/	75.64	75.56	64.96
	Gln185	25.90	/	87.90	90.08	58.52

Table S3. Normal-Coordinate structural decomposition of harderoheme during the rotation process of wild-type and Lys149Ala mutant.

	Basis	Doop	B2u	B1u	A2u	Eg(x)	Eg(y)	A1u
**WT**	**min**	0.81	0.38	0.31	0.30	0.09	0.35	0.44
	**max**	1.16	1.10	0.16	0.11	0.13	0.20	0.22
	**delta**	0.36	0.72	-0.14	-0.18	0.05	-0.15	-0.22
**Lys149Ala**	**min**	0.75	0.11	0.62	0.28	0.23	0.14	0.13
	**max**	1.63	1.59	0.03	0.22	0.06	0.15	0.18
	**delta**	0.88	1.48	-0.59	-0.07	-0.17	0.02	0.05

Table S4. Energetic change of enthalpy and entropy (kcal·mol^{-1}) by MM-PBSA of different reorientation process at each peak point.

	**Harderoheme clockwise**	**Harderoheme anticlockwise**	**Lys149Ala clockwise**	**Heme clockwise**	**Harderoheme releasing**	**Heme releasing**
ΔH	19.06	63.62	56.19	50.21	63.63	20.04
$T\Delta S$	4.03	10.76	6.54	19.42	18.05	1.69
ΔG	15.03	52.86	49.65	30.79	45.58	18.35

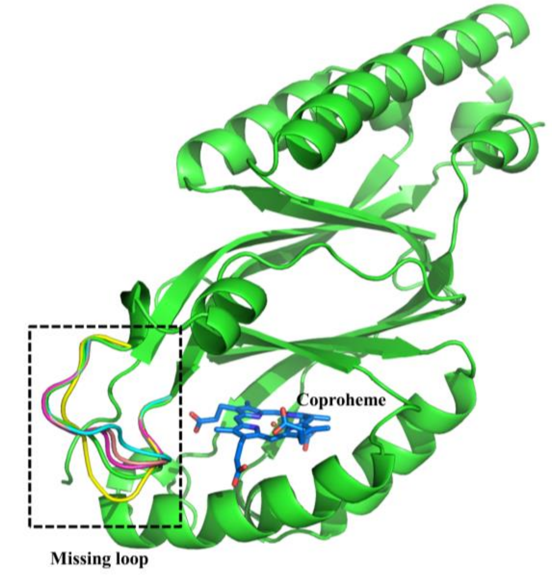

Figure S1. Initial model of five plausible completed chains by Modeller. The structures of FM1 (green), FM2 (cyan), FM3 (magenta), FM4 (yellow) and FM5 (wheat) were shown in cartoon.

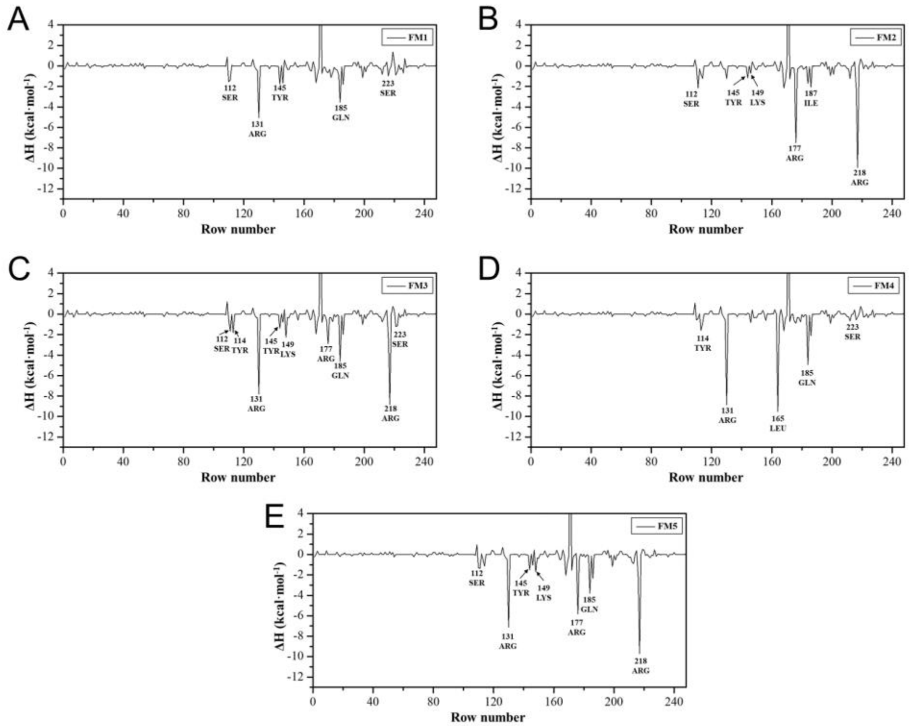

Figure S2. MM-PBSA energy decompositions representing (kcal·mol^{-1}) into the residues from FM1 to FM5 for five monomer models.

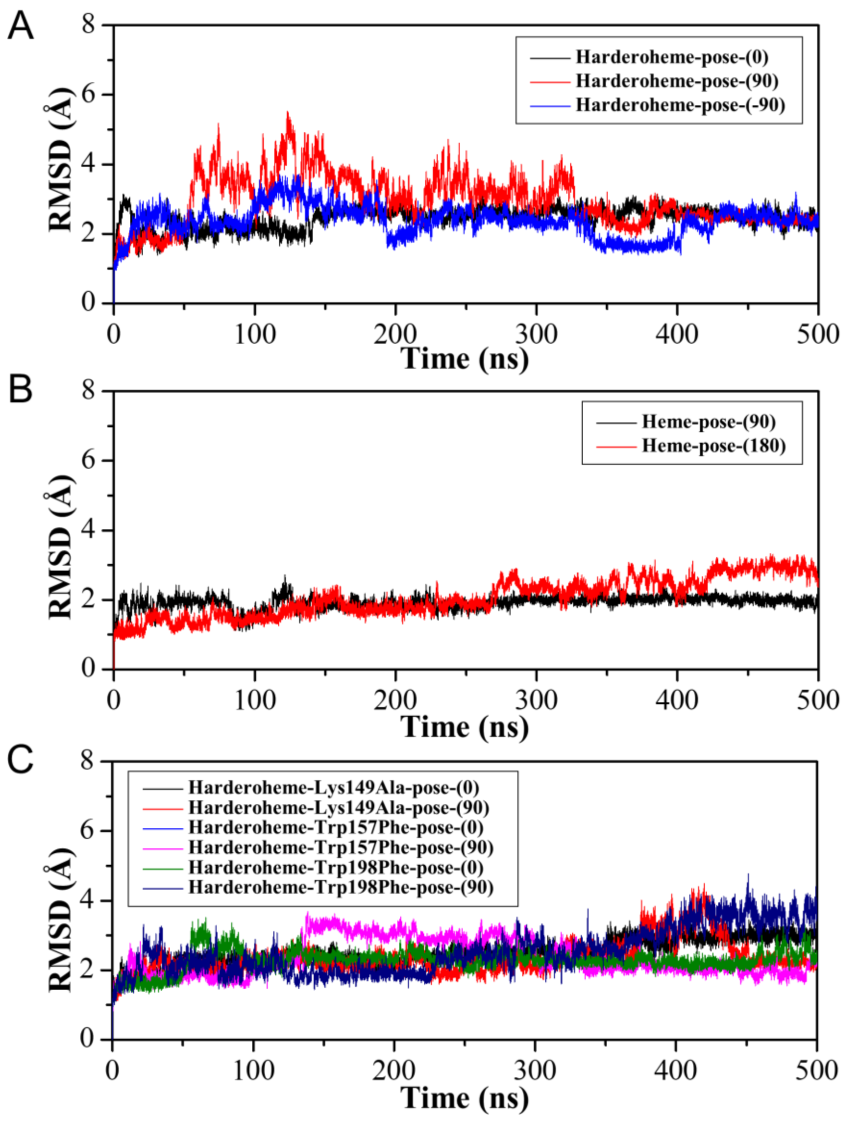

Figure S3. Root mean square deviation (RMSD) values of heavy atoms with respect to the initial structure of the TMD system: (A) Harderoheme-pose-(0) model in black line, Harderoheme-pose-(90) model in red line and Harderoheme-pose-(-90) model in blue line; (B) Heme-pose-(90) model in black line and Heme-pose-(180) in red line; (C) Harderoheme-Lys149Ala-pose-(0) model in black line, Harderoheme-Lys149Ala-pose-(90) model in red line, Harderoheme-Trp157Phe-pose-(0) model in blue line, Harderoheme-Trp157Phe-pose-(90) in magenta line, Harderoheme-Trp198Phe-pose-(0) model in green line and Harderoheme-Trp198Phe-pose-(90) model in deep blue line.

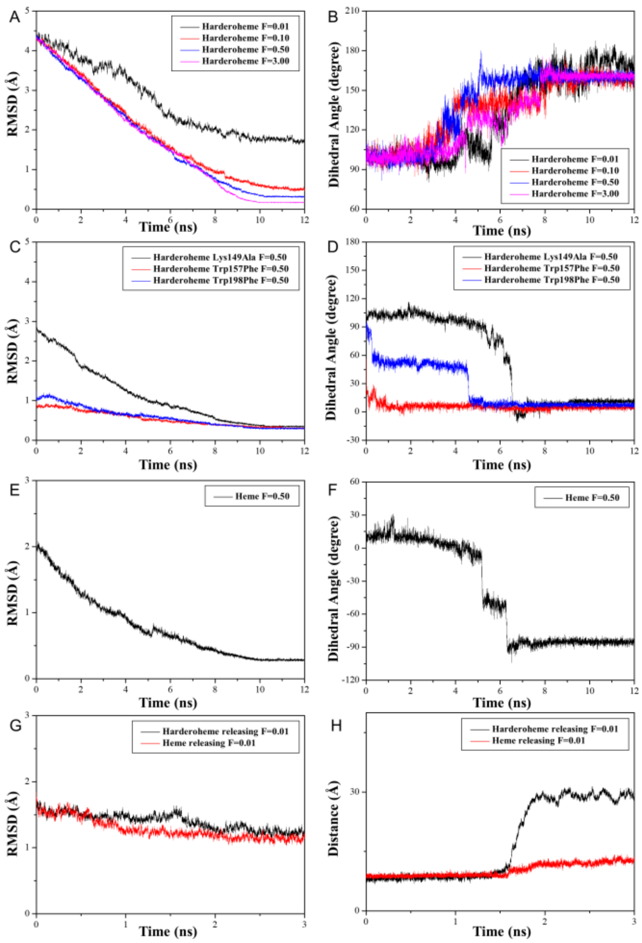

Figure S4. Root mean square deviation (RMSD) values of all backbone atoms and dihedral angle of $C_{a,\ sub\text{-}p4}$- Fe_{sub}- $N_{d,\ His171}$- $O_{h,\ Tyr145}$ from each targeted molecular dynamics (TMD) simulations corresponding by using different harmonic force constant of different process. (A) harderoheme anti-clockwise rotation process of harmonic force constant of 0.01 kcal/ (mol·Å2) in black, 0.10 kcal/ (mol·Å2) in red, 0.50 kcal/ (mol·Å2) in blue and 3.00 kcal/ (mol·Å2) in magenta; (C) Lysl49Ala clockwise rotation process of harmonic force constant of 0.50cal/ (mol·Å2) in black, Trp157Phe clockwise rotation process of harmonic force constant of 0.50cal/ (mol·Å2) in red and Trp198Phe clockwise rotation process of harmonic force constant of 0.50cal/ (mol·Å2) in blue; (E) Heme clockwise rotation process of harmonic force constant of 0.50 kcal/ (mol·Å2) in black. (G) Harderoheme releasing process of harmonic force constant of 0.01 lcal/ (mol·Å2) in red and Heme releasing process of harmonic force constant of 0.01 cal/ (mol·Å2) in red. The dihedral angle of $C_{a,\ sub\text{-}p4}$- Fe_{sub}- $N_{d,\ His171}$- $O_{h,\ Tyr145}$ of harderoheme clockwise rotation process.

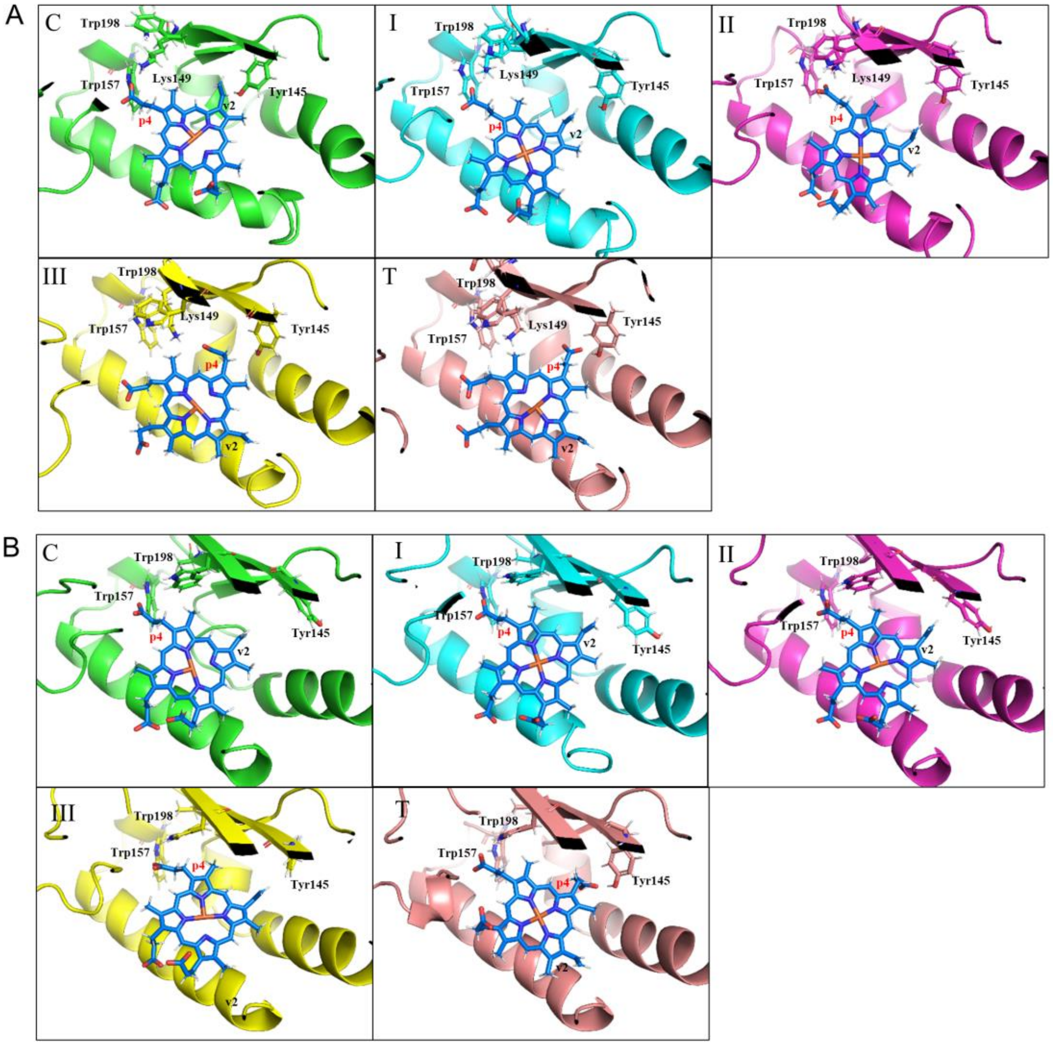

Figure S5. Top view of active site structures rotation process. Structure snapshots were shown in the corresponding color of each stage: (A) The current stage of WT model in green, stage I of WT model in cyan, stage II of WT model in magenta, stage III of WT model in yellow and the target stage of WT model in wheat cartoon; (B) The current stage of Lys149Ala model in green, stage I of Lys149Ala model in cyan, stage II of Lys149Ala model in magenta, stage III of Lys149Ala model in yellow and the target stage of Lys149Ala model in wheat.

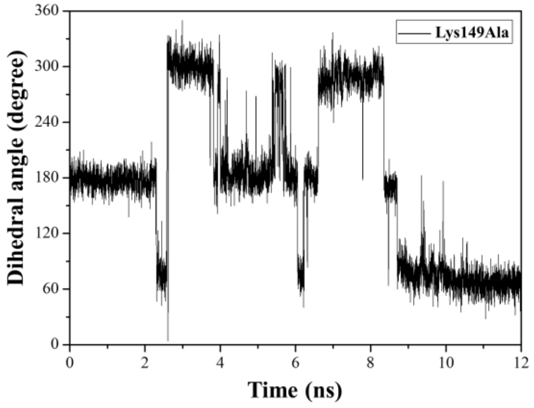

Figure S6. Dihedral angle of C_γ-C_δ-C_ε-C_ζ of Lys149 side-chain of the Lys149Ala mutant system.

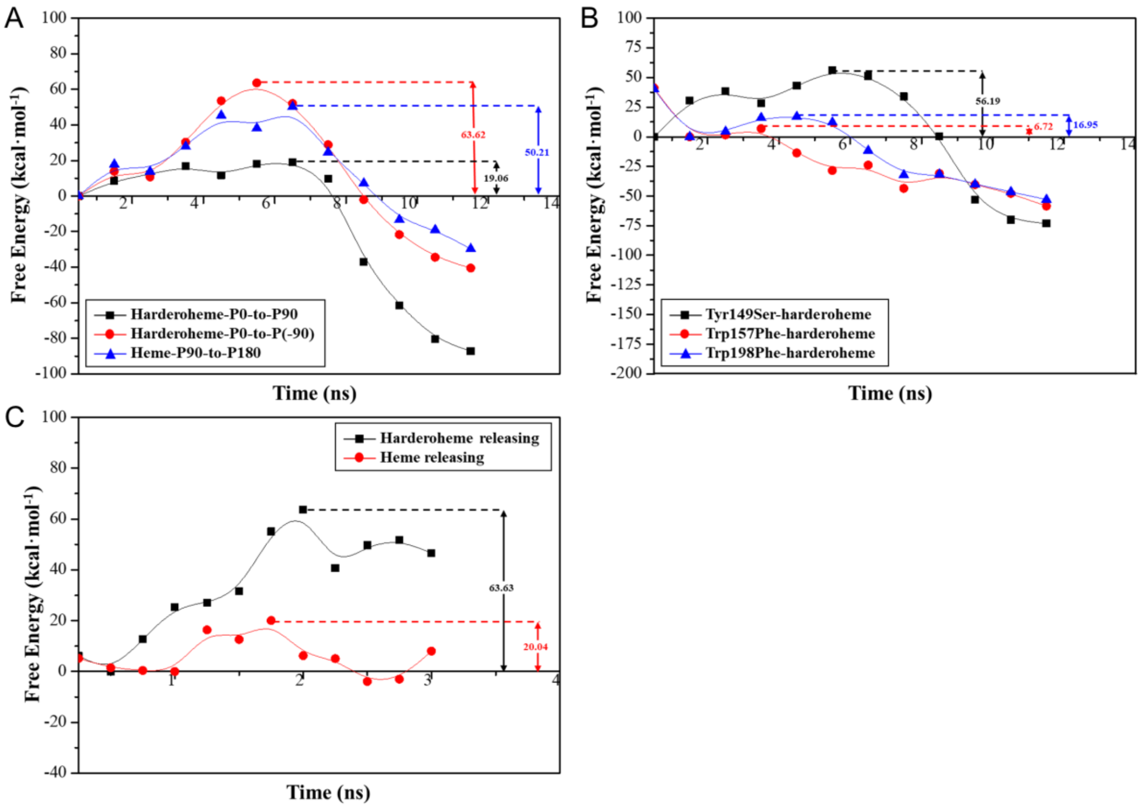

Figure S7. The calculated energy curve of enthalpy by MM-PBSA approach of (A) Harderoheme clockwise rotation process in black, Harderoheme anti-clockwise rotation process in red and Heme clockwise rotation process in blue; (B) Lys149Ala mutant clockwise rotation process in black, Trp157Phe mutant clockwise rotation process in black and Trp198Phe mutant clockwise rotation process in black; (C) Harderoheme releasing process and Heme releasing process.

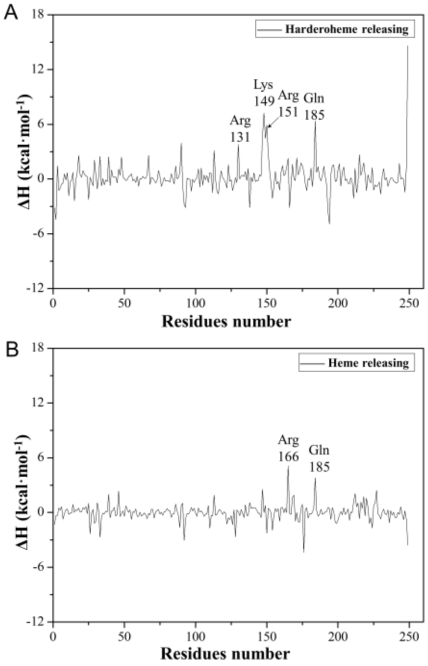

Figure S8. MM-PBSA energy decompositions of barriers into residues for different stages revealed by the TMD simulations harderoheme releasing process and (B) heme releasing process.

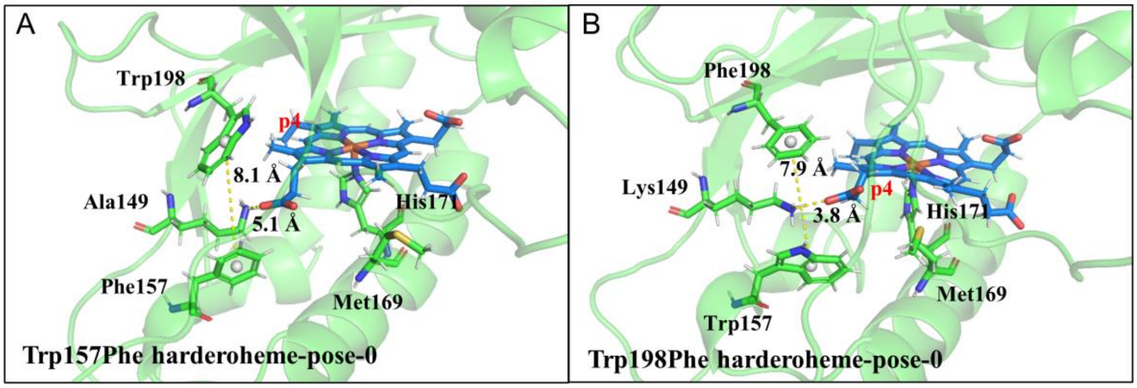

Figure S9. The structures after MD simulations of (A) Trp157Phe mutant of harderoheme-pose-0 and (B) Trp198Phe mutant of harderoheme-pose-0. Key distances among Trp-Lys-Trp portal and the substrate are shown.

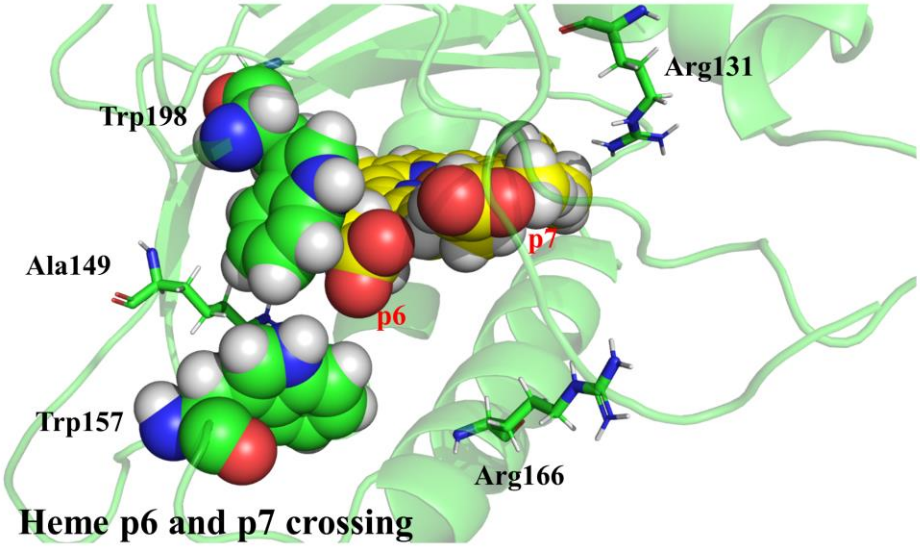

Figure S10. Representative active site structures at p4 crossing through the Trp157 and Trp198 residue of the harderoheme rotation process.

References

[1] Li, Chaoqun, *et al.* DNA structural distortions induced by a monofunctional trinuclear platinum complex with various cross-links using molecular dynamics simulation. *Journal of Chemical Information and Modeling*, 2020, 60(3): 1700-1708.

[2] Izaguirre, Jesus A., *et al*. Langevin stabilization of molecular dynamics. *The Journal of chemical physics*, 2001, 114(5): 2090-2098.

[3] Berendsen, Herman JC, *et al.* Molecular dynamics with coupling to an external bath. *The Journal of chemical physics*, 1984, 81(8): 3684-3690.

[4]. Ulrich E. *et al*. A smooth particle mesh Ewald method. *Int. J. Chem. Phys.*, 1998, **103,** 8577.

[5]. Miyamoto, S. & Kollman, P. A. Settle: An analytical version of the SHAKE and RATTLE algorithm for rigid water models. *J. Comput. Chem.*, 1992, **13,** 952-962.

[6]. Celis AI, Gauss GH, Streit BR, Shisler K, Moraski GC, Rodgers KR et al. Structure-Based Mechanism for Oxidative Decarboxylation Reactions Mediated by Amino Acids and Heme Propionates in Coproheme Decarboxylase (HemQ). J Am Chem Soc 2017;139(5):1900–11.

[7]. Gohlke, H., Kiel, C. & Case, D. A. Insights into Protein–Protein Binding by Binding Free Energy Calculation and Free Energy Decomposition for the Ras–Raf and Ras–RalGDS Complexes. *J. Mol. Biol.*, 2003, **330,** 891-913.

[8] Peherstorfer, Sandra, *et al.* Insights into mechanism and functional consequences of heme binding to hemolysin-activating lysine acyltransferase HlyC from Escherichia coli. *Biochimica et Biophysica Acta (BBA)-General Subjects*, 2018: 1862(9): 1964-1972

[9] Wißbrock, Amelie, *et al*. Structural insights into heme binding to IL-36α proinflammatory cytokine. *Scientific reports*, 2019, 9(1): 1-14.

[10] Hou, Xuben, et al. How to improve docking accuracy of AutoDock4. 2: a case study using different electrostatic potentials. *Journal of chemical information and modeling,* 2013, 53(1): 188-200.
